# Functional mechanical behavior of the murine pulmonary heart valve

**DOI:** 10.1038/s41598-023-40158-w

**Published:** 2023-08-08

**Authors:** Xinzeng Feng, Yifei Liu, David Kamensky, David W. McComb, Christopher K. Breuer, Michael S. Sacks

**Affiliations:** 1 Willerson Center for Cardiovascular Modeling and Simulation, Oden Institute for Computational Engineering and Sciences, The University of Texas at Austin, Austin, TX 78712 USA; 2 Department of Biomedical Engineering, The University of Texas at Austin, Austin, TX 78712 USA; 3 Center for Electron Microscopy and Analysis, The Ohio State University, Columbus, OH 43210 USA; 4 Department of Materials Science and Engineering, The Ohio State University, Columbus, OH 43210 USA; 5 Department of Mechanical and Aerospace Engineering, University of California San Diego, San Diego, CA 92093 USA; 6 Center for Regenerative Medicine, Abigail Wexner Research Institute, Nationwide Children’s Hospital, Columbus, OH 43205 USA; 7 Department of Pediatric Surgery, Nationwide Children’s Hospital, Columbus, OH 43205 USA

**Keywords:** Computational models, Valvular disease

## Abstract

Genetically modified mouse models provide a versatile and efficient platform to extend our understanding of the underlying disease processes and evaluate potential treatments for congenital heart valve diseases. However, applications have been limited to the gene and molecular levels due to the small size of murine heart valves, which prohibits the use of standard mechanical evaluation and in vivo imaging methods. We have developed an integrated imaging/computational mechanics approach to evaluate, for the first time, the functional mechanical behavior of the murine pulmonary heart valve (mPV). We utilized extant mPV high resolution *µ*CT images of 1-year-old healthy C57BL/6J mice, with mPVs loaded to 0, 10, 20 or 30 mmHg then chemically fixed to preserve their shape. Individual mPV leaflets and annular boundaries were segmented and key geometric quantities of interest defined and quantified. The resulting observed inter-valve variations were small and consistent at each TVP level. This allowed us to develop a high fidelity NURBS-based geometric model. From the resultant individual mPV geometries, we developed a mPV shape-evolving geometric model (SEGM) that accurately represented mPV shape changes as a continuous function of transvalvular pressure. The SEGM was then integrated into an isogeometric finite element based inverse model that estimated the individual leaflet and regional mPV mechanical behaviors. We demonstrated that the mPV leaflet mechanical behaviors were highly anisotropic and nonlinear, with substantial leaflet and regional variations. We also observed the presence of strong axial mechanical coupling, suggesting the important role of the underlying collagen fiber architecture in the mPV. When compared to larger mammalian species, the mPV exhibited substantially different mechanical behaviors. Thus, while qualitatively similar, the mPV exhibited important functional differences that will need to accounted for in murine heart valve studies. The results of this novel study will allow detailed murine tissue and organ level investigations of semi-lunar heart valve diseases.

## Introduction

Congenital heart valve disease (CHVD) affects more than 1% of all newborns in the United States. Overall, death rates due to CHVD have shifted away from infants and towards adults due to improved replacement valve delivery methods. However, there remain continued durability issues with replacement valves, with no current clinically available method that can accommodate patient growth^1,2^. Moreover, there are currently no non-surgical procedures to treat CHVD diseases, in large part because our understanding of its complex etiology remains incomplete. With respect to the broader study of human heart valve (HV) diseases, direct assessment in humans remains hampered by the fact that the data available is only at treatment, where the disease is at or near end stage, and time progression information is not available. Moreover, the clinical focus is usually replacement or repair, so the end-stage disease tissues and cells remain difficult to obtain. Alternatively, while utilized for many years for the assessment of replacement heart valve devices, large animal models remain unusable for the study of most heart diseases due to the inability to reproduce the key features of disease processes. Primate models have also been considered, but cost, logistics, ethical concerns, and lack of good disease emulation prevents their use. Mouse models are a versatile and efficient platform for the study of many human diseases due to the ability to generate large numbers of syngenetic animals, coupled with established techniques for manipulating their genome. Additionally, compared to large mammalian models, mouse models are more cost efficient, and their genome is much closer to that of a human compared to other small animals (e.g., zebra fish, chicken). These advantages make mouse models potentially very suitable for the study of heart valve diseases, including CHVD.

As the primary driver of HV organ-level responses to hemodynamic forces, the mechanical behavior of the valve leaflets are known to be dramatically altered in disease, including such mechanisms as calcification, leaflet thickening, or collagen remodeling^3–5^. However, current studies using mouse models are focused on the protein and genetic levels, as well as basic morphology^6,7^. In contrast to what is known for large animals and humans, critical tissue and organ level functional characteristics have been overlooked due to the very small size of the murine HV (diameter ∼ 1 mm). *As a result, mechanical evaluation methods for large mammalian species are not applicable to the murine HV*^8,9^*.* Existing evaluation approaches of murine HV leaflets have attempted various experimental approaches, such as micropipette aspiration and atomic force microscopy, to probe local mechanical behaviors^10,11^. Such technologies have provided valuable insights into the regional behaviors of the murine HV tissues. However, these approaches can only obtain limited metrics of stiffness (e.g., an effective Young’s modulus), which are not sufficient to characterize the complete, complex multi-axial mechanical behavior of the murine HV leaflet^12–14^. Similarly, it is also difficult to acquire high resolution images of a murine HV in vivo with adequate fidelity. While high-speed imaging techniques for murine HV exist^15–17^, these promising techniques are in a nascent stage. Moreover, they cannot provide the necessary resolution for highly detailed, quantitative studies necessary to establish baseline organ level information on murine HVs. Without such information, it is not currently possible to develop an accurate and robust understanding of how the murine HV remodels in disease, which significantly limits the use of murine HVs in the systematic study of HV disease.

In the present study, we developed an in vitro integrated imaging–computational approach to quantify, for the first time, the functional mechanical behaviors of murine pulmonary valve (mPV) leaflets from an extant series of ex vivo* µ*CT images^18^. An extant set of high resolution mPV *µ*CT images of 1-year-old healthy C57BL/6J mice were used, which consisted of mPVs loaded to 0, 10, 20 or 30 mmHg then chemically fixed to preserve their shape. Individual mPV leaflets and annular boundaries were segmented and key geometric quantities of interest defined and quantified. The resulting observed inter- valve variations in these quanties were small and consistent at each TVP level. This consistency in mPV geometry allowed us to develop, from the individual mPV geometries, a shape-evolving geometric model (SEGM) that accurately represented mPV geometry as a continuous function of transvalvular pressure. The SEGM was then integrated into an isogeometric finite element based inverse model that estimated the individual leaflet and regional mPV mechanical behaviors. We demonstrated that the mPV leaflet mechanical behaviors were highly anisotropic and nonlinear, with substantial leaflet and regional variations. The results of this approach were then compared to the equivalent responses of other large mammalian species.

## Results

### Deformation of mPV under hydrostatic TVP

**Figure 1:**
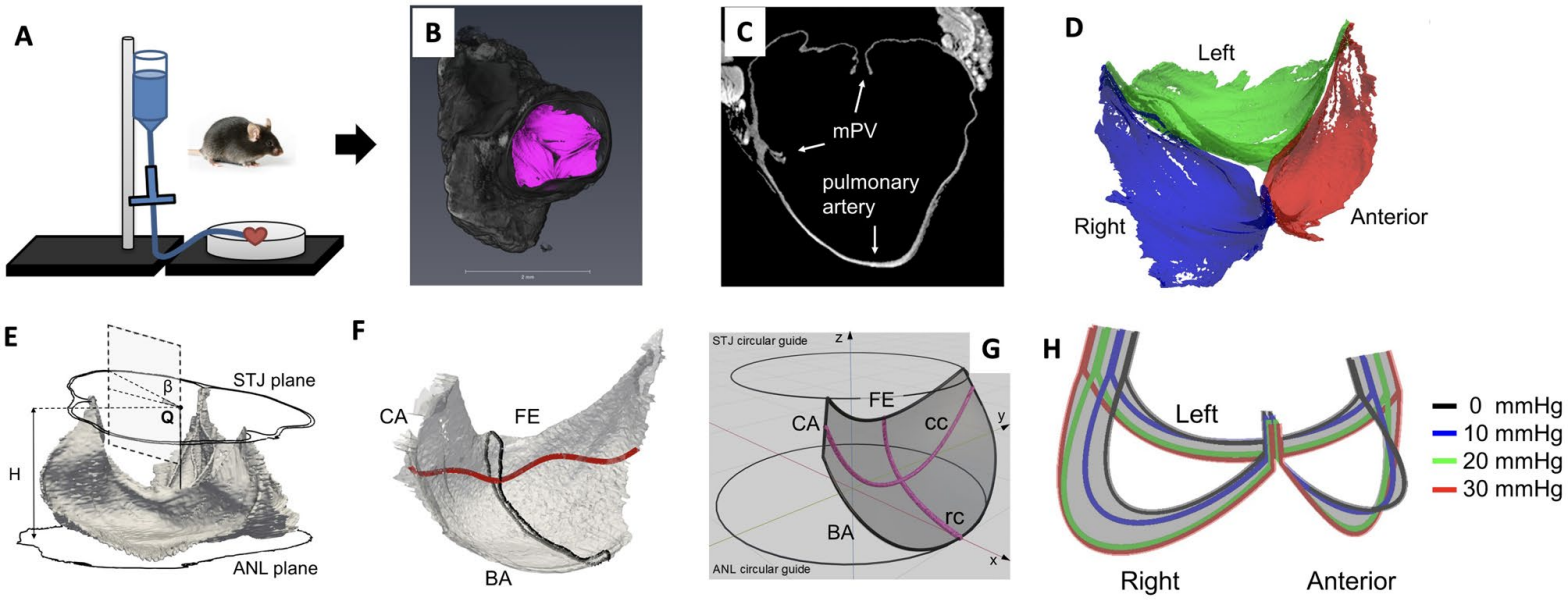
Schematic illustration of critical steps in developing the representative mPV geometries. (**A**) Each excised mPV was pressurized to set 0, 10, 20, or 30 mmHg, fixed, then (**B**) imaged with microCT. (**C**) A short-axis view from a raw *µ*CT image showing the mPV and the surrounding pulmonary artery. (**D**) An example of segmented mPV leaflets from *µ*CT images showing the anterior, right and left leaflets. (**E**) Selected gQOI on a segmented mPV geometry, where *β* was the tilt angle between the sino-tubular-junction (STJ) and annular (ANL) planes. **Q**, the upper center, was taken as the average position of the commissure points. (**F**) Central radial cross section (black) of a segmented leaflet and the estimate demarcation boundary (dark red) between the coaptation and belly regions (FE, BA, CA indicated the free edge, basal attachment and commissure attachment of the leaflet, respectively). (**G**) The generic geometric model (for a single leaflet shown only) and the associated central cross sections **cc***,* **rr** along the circumferential and radial directions. (H) Estimate root distention showing displacement of the basal attachment as TVP increased.

As a first step, we examined the geometric changes of the mPV underwent over a range of physiological transvalvular pressures (TVP) taken from^18^. Specifically, we utilized extant high resolution *µ*CT images of thirteen mPVs (Fig. 1A), each loaded hydrostatically to 0, 10, 20, and 30 mmHg. From the mPV segmented geometry (Fig. 1B–D), we defined and quantified geometric quantities of interest (gQOI) (Table 1). These gQOI included such quantities as the valvular height, tilt angle (Fig. 1E), and the extracted the cross-sectional profiles for each leaflet (Fig. 1F). Importantly, we have shown that these geometric characteristics exhibited a high degree of consistency between individual mPV at the same TVP^18^. This important feature of the mPV indicated that results from individual mPV could be combined. This is critical as the microCT imaging technique, while providing unique very high resolution 3D geometry, can only image a single mPV at a single TVP.

**Table 1:** Mean values of key gQOI at 0 mmHg showing the referential state geometry reasonably agreed with the direct measurement.

gQOI			Directly measured (0 mmHg)	Referential geometry	Error	% error
Valve height	H	Valve-level	N.A	523 µm	N.A	N.A
Annular perimeter^a^	L_p_	Valve-level	4455 µm	4433 µm	22 µm	0.5%
Tilt angle	β	Valve-level	N.A	9.2°	N.A	N.A
Free edge length	LFE	Left and right	1307 µm	1342 µm	35 µm	2.7%
		Anterior	1192 µm	1160 µm	32 µm	2.7%
Basal attachment length	LBA	Left and right	1505 µm	1496 µm	9 µm	0.67%
		Anterior	1446 µm	1441 µm	5 µm	0.4%
Commissure length^b^	LCA	Left and right	403 µm	392 µm	11 µm	2.7%
		Anterior	365 µm	354 µm	11 µm	3.0%
Leaflet surface area	a_*ℓ*_	Left and right	4.92 × 10^5^ µm^2^	4.90 × 10^5^ µm^2^	0.02 × 10^5^ µm^2^	0.4%
		Anterior	4.23 × 10^5^ µm^2^	4.25 × 10^5^ µm^2^	0.02 × 10^5^ µm^2^	0.4%

Due to leaflet collapse, valve height and tilt angle were not accessible from the segmented images, hence not included.

^a^L_p_ was defined as the sum of all basal attachments of a mPV.

^b^L_CA_ included the length of both commissure attachments of a leaflet.

**Figure 2:**
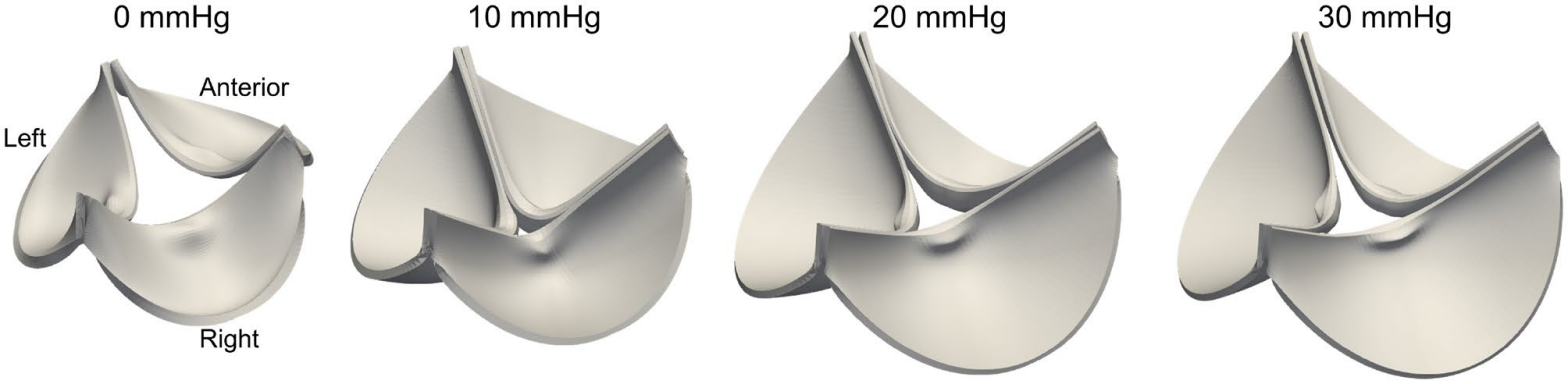
The referential state (0 mmHg) and representative mPV geometries at 10, 20 and 30 mmHg revealed the coherent deformation patterns of mPV in the ex vivo experiment. Volumetric rendering was created by incorporating average leaflet thickness measurement to the belly and nodulus regions.

We proceeded to integrate the geometric characteristics of individual mPVs into a single *representative geometric model* (SEGM) as a function of the TVP as follows. First, to describe the mid-surfaces of each leaflet and regularize the representative geometries, we developed a NURBS-based model which captured the major features in the segmented mPV geometry (e.g., symmetry between the right and left leaflets and the nonzero tilt angle^18^) (Fig. 1G). Next, we determined the distention of murine pulmonary root at different TVPs and used it to prescribe the displacement of the basal attachment in the representative geometric model (Fig. 1F). Results indicated that the developed SEGM mPV geometries (Fig. 2) agreed well with individual gQOI measurements (Table 1, Fig. S1), as well as the average mPV cross-sectional profiles at each TVP, suggesting a faithful representation of the *ex vivo* experiment. It also confirmed our assumption that individual mPVs, while loaded and imaged separately, demonstrated a consistent set of deformed geometries under increasing hydrostatic TVP. In the third step, we predicted the unloaded mPV geometry at 0 mmHg (i.e. the referential state geometric model). This was required, as the mPV root geometry was accurately recovered but leaflets tended to be distorted at 0 mmHg. Thus, the 0mmHg mPV leaflets were not directly usable to generate the unloaded configuration, although they were useful for several key dimensional measurements. We instead developed a referential state geometric model that matched all gQOIs accurately at 0 mmHg (Table 1 and Fig. S1). The reconstructed unloaded mPV geometry revealed detailed geometric features at 0 mmHg, such as a tilt angle approximately 9.2° between the sino-tubular junction (STJ) and annular (ANL) planes (i.e. a form a structural asymmetry).

### Systematic investigation of the regional mechanical behaviors of mPV leaflets

**Figure 3:**
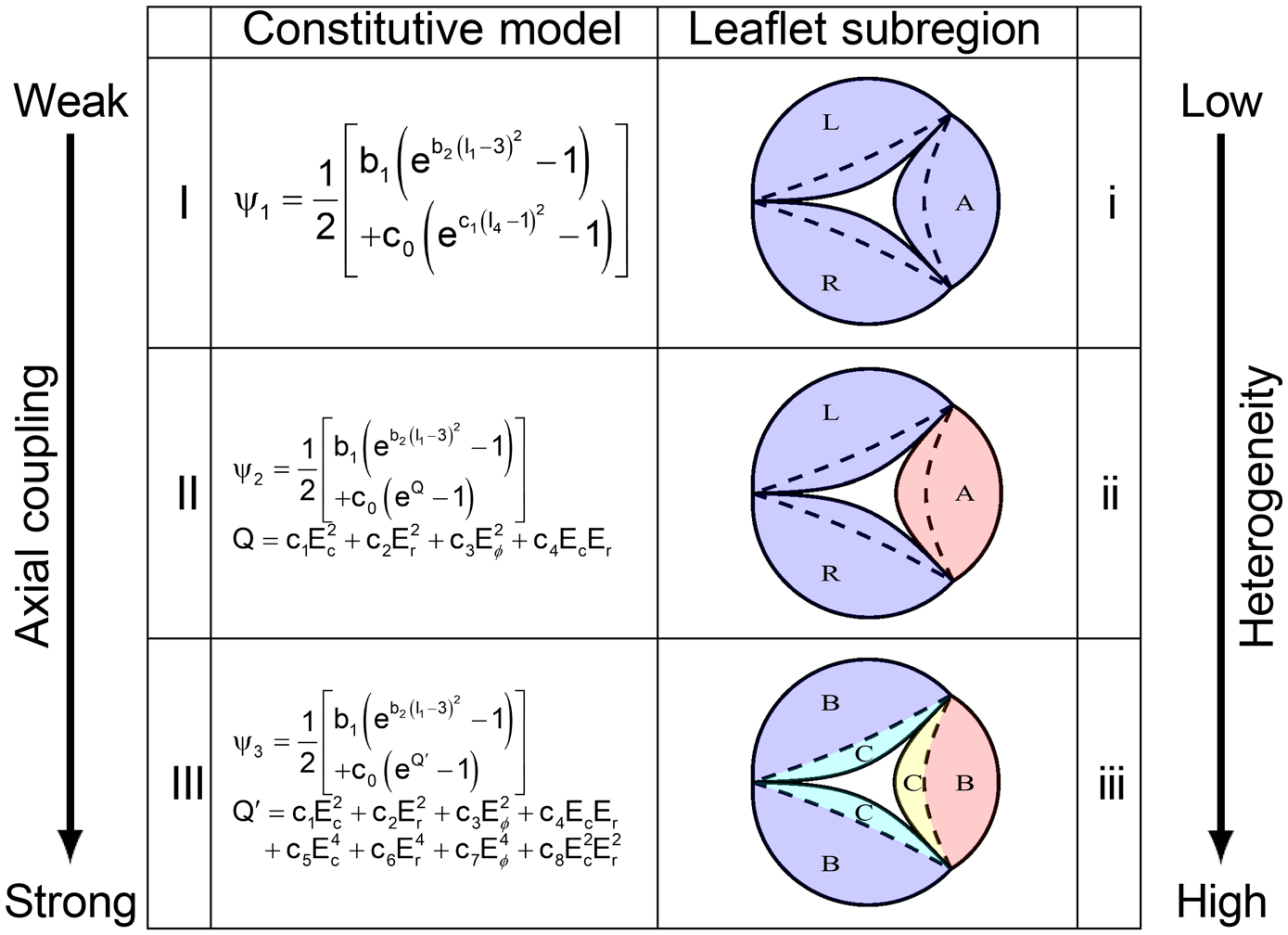
Model hierarchy to extract regional mPV mechanical behaviors. Different colors indicated leaflet subregions with different sets of constitutive model parameters. Respectively, A, R, L represented the anterior, right and left leaflets. B, C represented the belly and coaptation regions. Due to the right and left symmetry of mPV, same constitutive model parameters were assumed for the right and left leaflets. The degree of axial coupling that each model was capable of was increased by including additional explicit coupling terms, e.g. the *c*_4_ in Model II and *c*_4_ and *c*_6_ in Model III.

In this next step, we utilized the SEGM (Fig. 2) to extract the regional mechanical behavior of mPV leaflets using an inverse modeling approach. One issue that is not clear is the degree of regional intra- and inter-leaflet variations exist in the mPV, as reported for the larger mammalian species^12,13^. We thus utilised a sequence of increasingly complex constitutive models and separation of leaflet into sub-regions (Fig. 3). Each constitutive model consisted of an isotropic term to account for the ground matrix and an anisotropic term for the collagen fibers, with a major contribution along the circumferential direction of each leaflet^18^. The main difference in the models was how material axial coupling was modeled, as well known phenomena in heart valve tissues^12,13,19^. The sequence was a follows:

1. Model I, there was no explicit coupling in the anisotropic term (Eq. 1).
2. Model II introduced an intermediate level of coupling by including the product of the extensional components of Green–Lagrange strain tensor along the circumferential and radial directions (equa- tion 2).
3. Model III included higher order coupling terms (Eq. 3).

In addition to optimal material model identification, we also explored the potential presence of intra- and inter-leaflet regional variations (methods i, ii, and iii, Fig. 3). This was based on known sub-regional variations in semi-lunar heart valves present in larger mammals^12,13^. We started with assuming fully constant properties (Fig. 3, method i). This was followed by allowing the anterior leaflet to have separate material constants (Fig. 3, method ii). In the final model, we allowed both separate anterior leaflet and coaptation/belly regional variations (Fig. 3, method iii). This last model based in particular in the known belly/coaptation region variation reported in the aortic heart valve^12,13^. To quantify the most appropriate model, we utilize the normalized *ℓ*_2_ error between the representative mPV geometry at each TVP level and the simulated solutions derived from the five different combinations of constitutive models and leaflet subregion methods.

**Figure 4:**
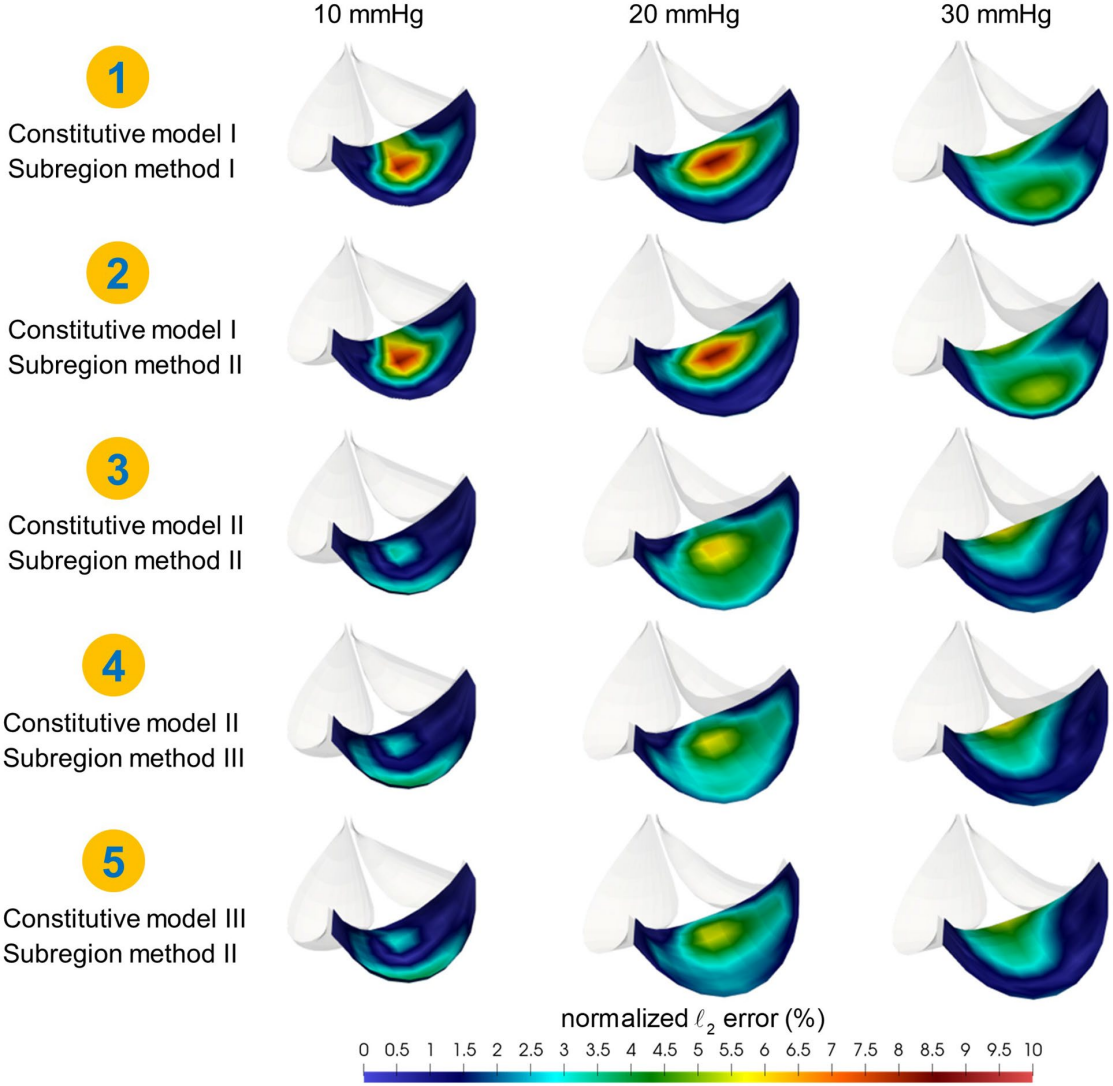
Normalized *ℓ*_2_ error of the simulated geometry against the representative mPV geometries at 10, 20 and 30 mmHg (shown for the right leaflet). The simulated geometry was generated based on the optimal solutions using a hierarchy of schemes combining different constitutive models and leaflet subregion methods. From scheme I—i (row 1) to scheme III—ii (row 5), model complexity gradually increased.

The resulting simulation results indicated that increasing axial coupling in the constitutive model substantially reduced the *ℓ*_2_ error (Fig. 4). In particular, the maximum value of the normalized *ℓ*_2_ error was found to decrease noticeably from material model I to model II, especially at 10 and 20 mmHg (see schemes I—ii, II—ii). From model II to model III, a slight decrease was observed at the central belly region at 20 mmHg from about 4% to 2% (see schemes II—ii, III—ii). On the other hand, partitioning each leaflet into the coaptation and belly regions also substantially improved the fitting performance but in a less significant manner. For constitutive model I, the difference in the *ℓ*_2_ error was insignificant between schemes I—i and I—ii. For model II (see schemes II—ii, II—iii), only subtle differences occurred at 20 mmHg when the normalized *ℓ*_2_ error decreased from 4 to 3% in the central belly region, and from 6 to 5% in the central coaptation region. Overall, we conclude that for mPV leaflets a proper choice of constitutive model and regional variations was crucial to accurate mechanical behavior estimations.

### Functional mechanical behaviors of mPV

**Figure 5:**
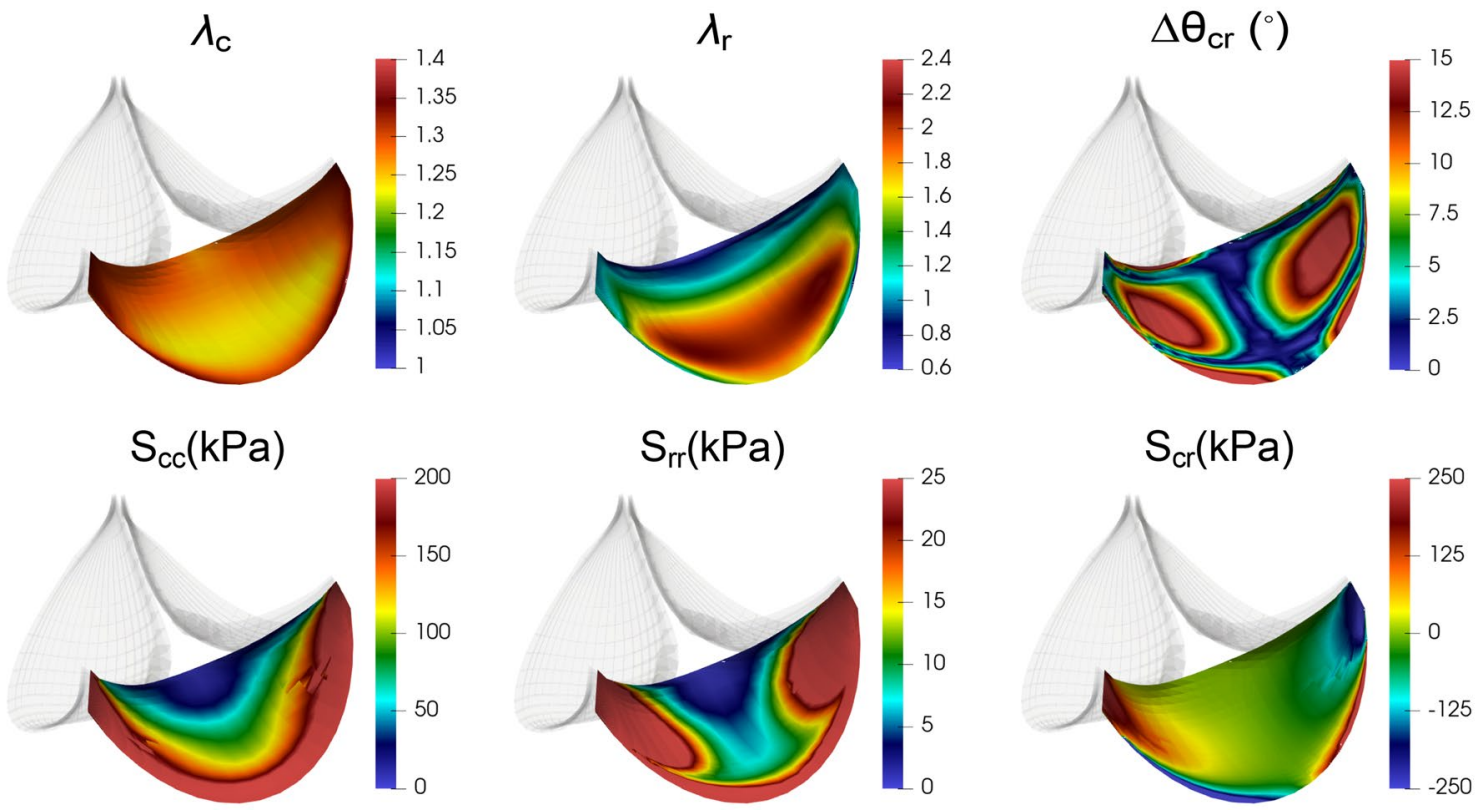
The simulated mPV geometry at 30 mmHg based on the optimization scheme II—iii. Here, *λ*_c_*, λ*_r_ were normal stretch ratios along the circumferential and radial directions. ∆θ_cr_ was the angle change between the circumferential and radial directions with respect to the referential state. And S_cc_*,* S_rr_*,* S_cr_ were in-plane components of the second Piola–Kirchhoff stress tensor.

Once the final constitutive model and regional variations were established, we then examined the resultant simulation results in detail. In particular, at the maximum TVP of 30 mmHg it was found that the magnitudes of the normal stress components S_cc_*,* S_rr_ were larger along the basal attachment compared to the central (nodulus) region, probably caused by the root distention (Fig. 5). The maximum shear angle was only about 15ׄ° and seen mostly near the basal and commissure attachments. This suggests that the very little shearing occurs in the mPV relative to the (presumably) local circumferentially aligned collagen fiber network. That is, the leaflets deform parallel to the circumferential and radial directions. Additionally, we noticed that there was little change in the in-plane stretch ratios *λ*_c_ and *λ*_r_, as well as the angle change ∆θ_cr_ from past 20 mmHg (Figs. S2, S3). This suggested that “locking” of collagen fibers in mPV leaflets likely occurred around or before 20 mmHg. In the belly region, the locking circumferential and radial stretches were estimated to be 1.25 and 2.2, respectively.

### Simulated mechanical response of the mPV under planar biaxial loading

**Figure 6:**
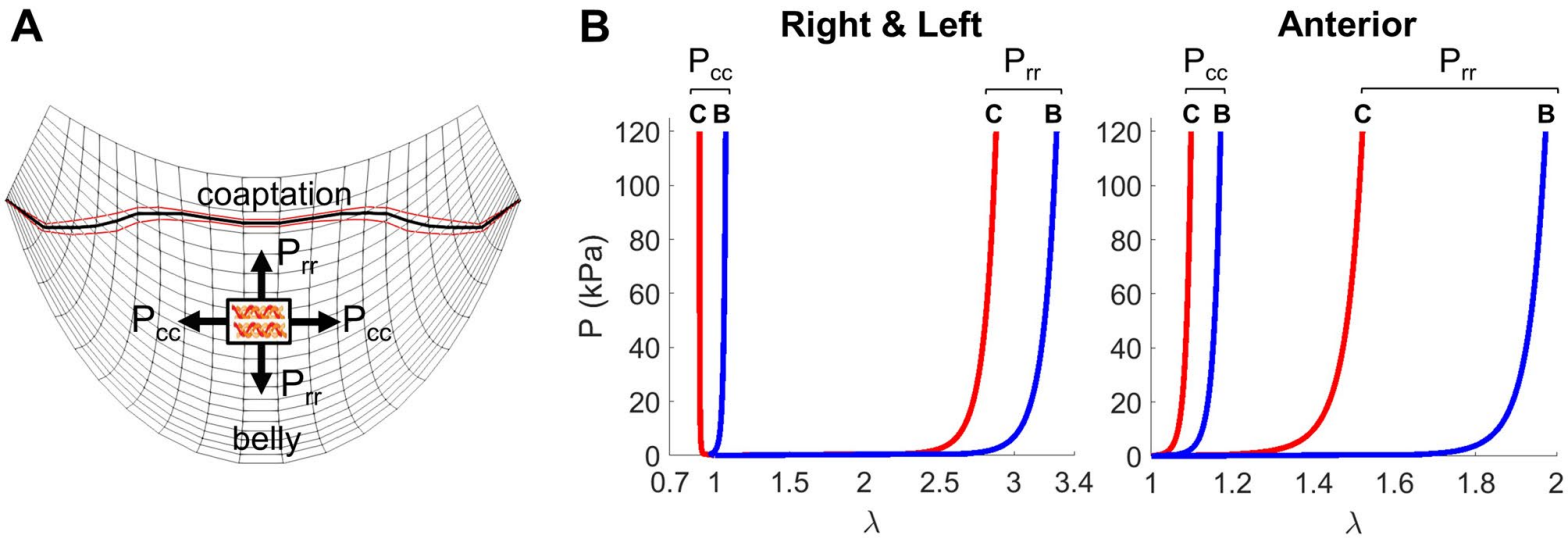
(**A**) Illustration of the coaptation and belly regions in a mPV leaflet to which different constitutive model parameters were assigned. Solid black curve represents the mean and the red curves 1 standard deviation of the boundary between the belly and coaptation regions, taken from the measured microCT data. Light black grid shows the local circumferential and radial directions. Also shown is a schematic of the small region used to simulate the planar biaxial responses (coaptation region not shown for clarity). (**B**) Resultant planar biaxial simulations showing the responses of the coaptation (red) and belly (blue) regions in the circumferential and radial directions for the right and left and anterior leaflets. While the right and left leaflets exhibited some regional variations, the anterior leaflet was much more pronounced.

**Figure 7:**
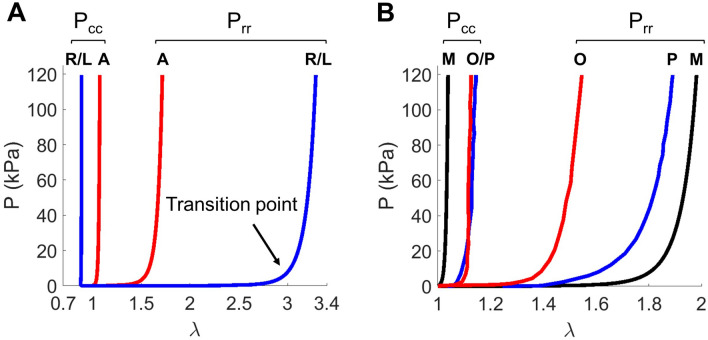
(**A**) Averaged mechanical responses (i.e. mean responses of the belly and coaptation regions) for the anterior leaflet (red) and the right and left leaflets (blue) under simulated planar biaxial loading. The greater anisotropy of the anterior leaflet was quite evident. (**B**) Comparison of the average mechanical responses of mPV leaflets (black) with porcine (blue) and ovine (red) data. The mPV was more highly anisotropic than the porcine or ovine PVs.

To gain further insights into the *intrinsic* mechanical behavior of the mPV leaflets, we *simulated* the responses of small square region mPV from the belly and coaptation regions of all leaflets under planar biaxial loading using the optimal constitutive model parameters (derived from scheme II—iii, see Supplementary Table S2) (Fig. 6A). All mPV leaflets were found to exhibit a stiffer response in the circumferential direction, while simultaneously more compliant initially in the radial direction, followed by a stiffer response (Fig. 6B). This behavior was presumably due to locking of collagen fibers. The anterior leaflet also exhibited more less mechanical anisotropy in the coaptation region, which was quite distinct from its belly region. Next, we averaged the mechanical responses (i.e. mean responses of the belly and coaptation regions) for the anterior leaflet (red) and the right and left leaflets (blue) under simulated planar biaxial loading. The greater anisotropy of the anterior leaflet was made quite evident (Fig. 7A).

Due to the unique nature of mPV, we next compared results from the present study with other studies of larger mammalian species. We found that mPV leaflets exhibited greater anisotropy than their porcine^20^ and ovine^21^ counterparts, with a stiffer response in the circumferential direction and a more compliant response in the radial direction (Fig. 7B). *To conclude, we demonstrate that mPV leaflets were highly nonlinear, with noticeable regional variations and exhibited greater anisotropy compared to those from large mammalian species.*

## Discussion

### Approach

Our goal in the present study was to estimate, for the first time, the mechanical behavior of the mPV leaflets. Unlike the larger mammalian heart valves, its very small size prohibits the use of standard mechanical evaluation and *in vivo* imaging methods. To this end, we developed an computational geometric/inverse mechanics modeling approach, based on a set of mPV microCT images acquired in an ex-vivo preparation at ranges of TVP. This was made possible in part due to high inter-specimen geometric regularity observed for the mPV^18^. To circumvent the inability to acquire the geometry from only one TVP for each mPV, we developed a detailed NURBS-based SEGM for the entire physiological TVP range (Fig. 2). This geometric model allowed us to accurately interpolate the geometric changes the mPV underwent with TVP. We then integrated this geometric model into an IGA-based finite element-based inverse computational mechanics model. Using a series of progressively more detailed material models and regional variations, we established for the first time the intra- and inter-leaflet hyperelastic mechanical behaviors of mPV leaflets.

### Key findings

We first demonstrated that to describe mPV leaflet hyperelastic mechanical behaviors, a material model with substantial axial mechanical coupling was required (model III, Fig. 3). This strong axial mechanical coupling is very consistent with other larger mammalian heart valve responses^12,13^, wherein the *rotation* of constituent collagen fibers induces such features as contraction in the circum- ferential direction. This has been shown to be a direct result of the circumferentially aligned collagen fibers^12,13,22^. While a detailed description of the collagen architecture in murine heart valves is not yet available, these observations are consistent with our previous SEM imaging results which indicated that local collagen fibers in a mPV leaflet had a mean orientation along the circumferential direction, with noticeable level of dispersion^18^. Although these results were only available from a small number of select points in the mPV, from a modeling perspective this suggested that the resulting axial coupling induced by a disperse collagen fiber orientations distribution should not be neglected or over-simplified in the leaflet material models.

We studied regional heterogeneity in two ways (Figs. 3, 6 and 7) and consistently found that inclusion regional variations (Model III) was needed to obtain the best agreement between the FE model and the deformed mPV geometry (Fig. 4). Interestingly, we found that the coaptation region was stiffer than the belly region, potentially caused by its associated increased collagen content^23^. More significant variation in the mechanical responses was observed among the leaflets suggesting different collagen architecture and properties in the anterior leaflet. Remarkably, we also found that mPVs had greater anisotropy compared to their counterparts in large mammalian animals (Figs. 6, 7). The reasons for these differences remain unknown, but are likely a direct result of the tissue and organ level specializations of the murine heart valve to their extremely high heart rates (600 bpm). While this has been shown to affect aortic root hemodynamics^24,25^, related studies on the murine heart valves remains largely unexplored. Overfall, the present results indicated that the mPV leaflets were highly nonlinear and exhibited substantial regional variations (Fig. 6), and that distinct regional variations in the mPV exist. However, future structural studies will be needed to allow for more detail structural modeling of the murine heart valves in health and disease.

### Use of an integrated imaging/computational approach

Our integrated imaging/computational approach demonstrated several advantages for understanding the functional mechanical behaviors of the mPV, as compared to limited one-dimensional metrics of stiffness from local evaluation approaches. First, there was no need to cut the mPV leaflet (diameter ∼ 1 mm) into smaller pieces and to mount the excised tissues on a mechanical testing device, which is technically not currently possible. Second, it allowed estimation of the mechanical behaviors for the entire leaflet (which are highly nonlinear, anisotropic, and exhibit regional variations) under physiological loading conditions. We have shown for the mitral^26^ and and aortic heart^27^ valves that they operate with substantial pre-strains. Such pre-strains can have a substantial impact on the organ level function and estimate of tissue properties and effects on constituent valve cellular behaviors^28–30^. Thus, our study underscores the both the need and an approach to evaluate mechanical function of valvular structures closer to their native functional environment.

### Limitations

Data from the present study was taken from an *ex vivo* experimental approach, wherein individual mPVs were first loaded then fixed at specified TVP. While it would have been ideal to perform a complete set of TVPs on the same valve, this was not possible using the present methods. This was not a critical issue as we have shown that the individual mPVs had highly consistent geometric characteristics^18^, and facilitated integration of the responses of multiple mPV to obtain improved population estimates. The resulting information on murine heart valve behavior is comprehensive and potentially allows direct connection to quantitative morphology from the fixed valves^18^. However, in a study of a disease process it would conceivably require a set of approximately 10 valves for each time point studied. Given the lower cost and relative ease of use in murine models, this should not be prohibitive, but does add time to study. We also observed that the mPV did not fully close at 30 mmHg (Fig. 2), due to the greater root distention that occurred in the *ex vivo* experimental approach. However, this did not limit our ability to extract the mechanical behaviors of the mPVs, as the leaflets were loaded under near physiological conditions. Finally, we note that we only compared three candidate phenomenological constitutive models. In the future, it would be worthwhile to investigate other more realistic constitutive models, e.g., the micro-structural or multiscale models^31–33^, as the requisite data becomes available.

### Future directions

Our study demonstrated the presence of strong material axial coupling in the mPV, as shown for the porcine aortic heart valve^12,13^. These fiber level processes in diseased conditions can potentially modify the properties of the mPV leaflets, and eventually cause the organ-level mechanical behaviors to become dysfunctional. This makes genetically-modified murine models with HV diseases a relevant platform to understand the etiology of murine HV diseases. Indeed, *via* genetic manipulation, it can potentially allows us to correlate the organ level disorders with lower level changes in a highly controlled manner. In the long term, we hope this work will facilitate the adoption of murine models as an efficient platform for testing pharmaceutical therapies to treat congenital heart valve diseases. The generalized technology can be eventually extended to *in vivo* once 3D imaging technologies for the murine heart valves become possible.

## Methods

**Figure 8:**
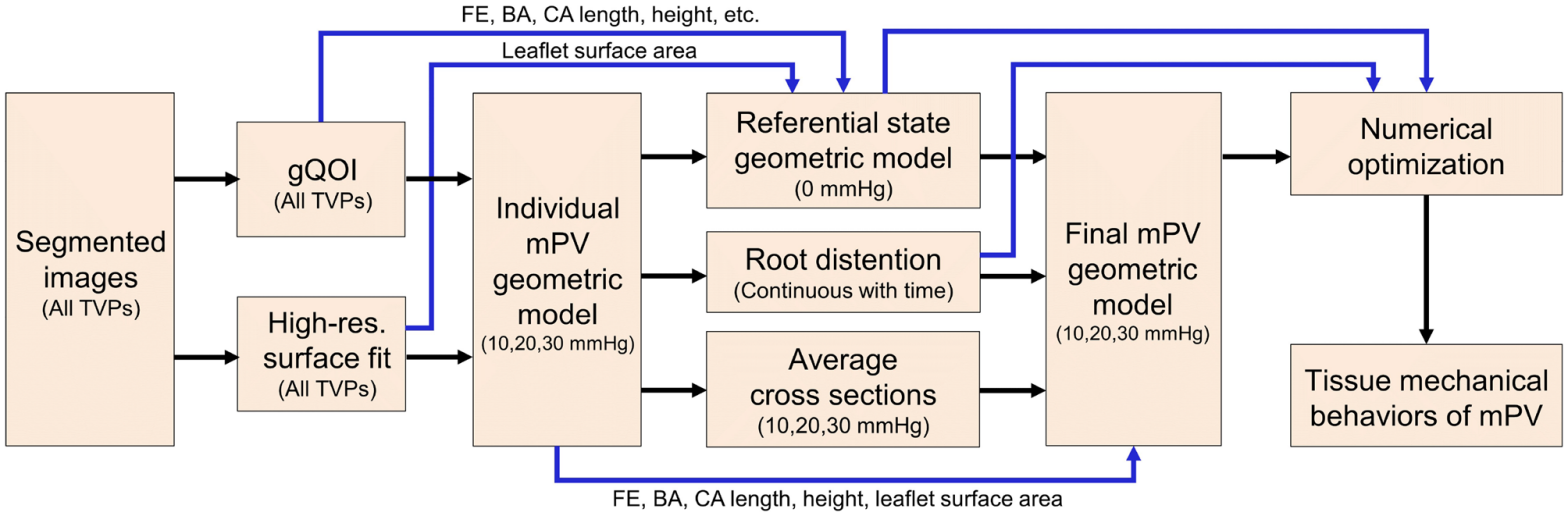
Analysis pipeline for developing the representative mPV geometric model and extracting the mechanical behaviors of mPV leaflets. In the first step, we obtained geometric characteristics of the segmented images via a list of gQOI and leaflet cross-section profiles which were derived from a high resolution NURBS surface fit for each leaflet. In the second step, we developed a regularized geometric model for each mPV based on the acquired geometric features. In the third step, we developed the referential state geometric model at 0 mmHg, estimated the root distention and derived the average mPV cross-sectional profiles for each TVP. In the fourth step, we integrated all these geometric information into the final representative geometric models at 10, 20 and 30 mmHg. In the last step, we used an inverse modeling framework to determine the tissue mechanical behaviors of mPV in different leaflets and leaflet subregions.

The following sections described the methods necessary to repeat the analysis pipeline (Fig. 8). More detailed procedure may be provided in the Supplementary Information online.

### Data acquisition

We used extant *µ*CT images of the pulmonary heart valves from thirteen healthy C57BL/6J mice at varying physiological TVP^18^. Each mPV (n = 2 at 0 mmHg, n = 3 at 10 mmHg, n = 5 at 20 mmHg, n = 3 at 30 mmHg) was pressured hydrostatically, chemically fixed, and then imaged using a Heliscan *µ*CT instrument (Thermo Fisher Scientific, resolution ∼ 5 µm). Pressurization was achieved by elevating a reservoir of solution to a height relative to the mPV. The polyethylene pressure monitor tubing was anastomosed to the pulmonary trunk using polyamide sutures. After the TVP was confirmed, fixing solution was gradually incorporated over 3h to fix the mPV conformation. The study was approved by Nationwide Children’s Hospital institutional animal care and use committee under protocol AR13-00030. All experiments performed on mice were done in accordance with relevant guidelines and regulations of this committee as well as the ARRIVE guidelines.

### Dimensional characterization

Segmentation of raw *µ*CT images was performed manually using Synopsys’s SimplewareTM ScanIP (Version 2018.12-SP2; Synopsys, Inc., Mountain View, USA) to separate the anterior, right and left leaflets (Fig. 1A,B). From the segmented geometry, a list of gQOI were measured (Fig. 1C, Supplementary Fig. S1) including the valve height (H), the length of the free edge (L_FE_), basal attachment (L_BA_) and commissure attachment (L_CA_) of each leaflet, the tilt angle (*β* ) between the annular (ANL) and sino-tubular-junction (STJ) planes, the distance between commissure points (d_comm_), distance between the leaflet noduli (d_nod_), leaflet thickness at the belly (w_belly_) and nodulus (w_nod_) regions, and leaflet surface area (a_*ℓ*_). The annular perimeter length (L_p_) was defined as the sum of all basal attachment lengths. The total attachment length of a leaflet was defined as the sum of basal and commissure attachment lengths. Since a segmented geometry was often subject to missing voxels, NURBS surface fit (see below) was used to estimate the leaflet surface area.

### Shape analysis

Nonuniform rational B-splines (NURBS) is a widely-used mathematical tool in computer-aided design that offers flexibility and accuracy to represent complex geometries. We used NURBS to construct a smooth, high-fidelity NURBS representation for the midsurfaces of individual mPV leaflets. Each NURBS surface was defined by two knot vectors, a grid of control points and associated weights. By optimizing the position of control points while keeping the knot vectors and weights fixed, we fitted a NURBS surface to a point cloud downsampled from the segmented leaflet geometry^18^. Given the NURBS representation, we acquired circumferential and radial cross sections of the leaflet by mapping isolines of the NURBS parametric domain onto the 3D geometry. All cross-sectional profiles were then projected onto the nearest vertical plane to allow for further averaging across different mPVs.

### Generic geometric model

A generic geometric model was developed to describe the closed-state mPV geometry. It was inspired by the rich literature of bioprosthetic heart valve design^34–38^, but extended to describe key features of a natural mPV including (1) right and left symmetry of the mPV, (2) nonzero tilt angle between the ANL and STJ planes, and (3) trefoil-shaped root projection on the ANL plane. The geometric model was constructed in a hierarchy. The base model assumed zero tilt angle and circular root projection. The full-feature model extended the base model with a sequence of transformations to introduce the nonzero tilt angle and trefoil-shaped root projection (see Supplementary Methods S1, Supplementary Fig. S4 and Table S3).

### Individual mPV geometric model fit

To construct individual mPV geometric models, we used the generic geometric model to fit the extracted dimensional and shape information of each mPV. The cost function included a weighted sum of total mismatches in the central cross-sectional profiles and key gQOI between the fitted and actual geometry (see Supplementary Methods S1 online). As suggested by the *µ*CT images, right and left leaflets of a mPV were largely symmetric with the anterior leaflet being slightly smaller^18^. Therefore, in all geometric models, right and left symmetry was assumed.

### Referential state geometry

The referential state geometry was determined in a way similar to the individual geometric models. The difference was that cross-sectional profiles as well as H*,* d_nod_*,* d_comm_ were not available at 0 mmHg due to leaflet collapse. As a result, only a partial list of gQOI at 0 mmHg were used to characterize the referential state geometry including the annular perimeter length, free edge length, basal/commissure attachment lengths (directly measured) and leaflet surface area (see Supplementary Methods S1 online). To improve the fidelity of reconstructed geometry, the fitted geometric parameters from individual mPV geometric models at 10, 20 and 30 mmHg were extrapolated to 0 mmHg as initial-guessed values. Then a slightly larger bracket were chosen to constrain the geometric parameters. The final fitted gQOI were summarized in Supplementary Table S1.

### Root distention

Distention of murine pulmonary root was assumed to be a smooth, spatially non-uniform vector field that varied monotonically w.r.t TVP. To avoid over-fitting, only a limited number of geometric parameters were introduced including the radial displacement at midpoint of the basal attachment and at the commissure point as well as the vertical displacement at midpoint of the basal attachment for each leaflet (see Supplementary Methods S1 and Supplementary Fig. S5 online). These parameters were used in a mathematical expression to define the displacement at any point on the total attachment and TVP between 0 and 30 mmHg. We determined these geometric parameters by fitting to selected gQOI at 10, 20 and 30 mmHg derived from individual mPV geometric models. Particularly, for the anterior leaflet, the middle point of the basal attachment experienced an inward 140 µm radial displacement and a downward 140 µm vertical distention from 0 to 30 mmHg. The right and left leaflets distended in both radial and vertical directions with magnitude around 200 and 140 µm, respectively (Fig. 1).

### Representative geometry

The representative geometry integrated three pieces of derived information: (1) the referential state geometry, (2) the root distention model, and (3) the average cross sections derived from individual mPV geometric models. Particularly, we incorporated the estimate root distention in the referential state mPV geometry by displacing the total attachment accordingly. The geometric parameters were then optimized similar to the process for developing the individual and referential state geometry (see Supplementary Methods S1 online) with two major differences. First, since the boundary of the geometric model was determine by the root distention model, related gQOI on the total attachment (i.e., L_p_*,* L_BA_*,* L_CA_) were matched automatically. Hence, they were excluded in the cost function. Second, average cross-sectional profiles among different mPVs at the same TVP were used to characterize the shape of the geometry. Despite that the representative mPV geometry may not represent the actual mPV deformation in vivo. In fact, due to removal of the ventricular tissues surrounding the mPV, the pulmonary root was less constrained leading to a slightly increasing orifice area from 10 to 30 mmHg. Nevertheless, this minor artifact did not undermine our ability to extract the mechanical behavior of mPV leaflets within the physiological range of TVP.

### Numerical simulation of the quasi-static state and optimization

#### Numerical setup

We modeled each leaflet as an incompressible hyperelastic Kirchhoff-Love thin shell with unloaded geometry dictated by the referential state mPV geometry. Using isogeometric analysis (IGA), we numerically computed the closing of mPV as the TVP increased quasi-statically. Leaflet-leaflet contact was implemented by defining a symmetry plane between each pair of leaflets. The contact potential was then computed based on the distance of a leaflet to the symmetry planes to avoid penetration^39^. To improve the stability of simulation, additional viscous and mass damping terms were introduced which essentially converted the quasi-static problem to a dynamic problem. As long as damping was sufficient, the solution of the dynamic problem converged to the equilibrium displacement with improved stability. Implementation of the numeric solver was done using the state-of-the-art finite element and shell analysis libraries in Python including FEniCS, tIGAr and ShNAPr^39^. Average thickness measurement was assigned to the belly (29 µm, uniform) and the nodulus region (100 µm at the widest). Compared to traditional finite element approaches, IGA is advantageous in parametric studies when different mPV geometries can be generated easily by varying the control point positions. It has also proved efficient for shell simulation^39^.

#### Material (Constitutive) model formulations

The strain energy density for constitutive model I was given by^40^

1 $$\psi_{1} = \frac{1}{2}\left[ {{\text{b}}_{1} \left( {\exp \left[ {{\text{b}}_{2} \left( {{\text{I}}_{1} - 3} \right)^{2} } \right] - 1} \right) + {\text{c}}_{0} \left( {\exp \left[ {{\text{c}}_{1} \left( {{\text{I}}_{4} - 1} \right)^{2} } \right] - 1} \right)} \right]$$

in which I_1_ = tr(**C**) was the first invariant of the right Cauchy Green’s deformation tensor **C** = **F**^T^**F** where **F** was the deformation gradient, I_4_ = **e**_c_ **Ce**_c_ was the fourth invariant of **C** along the circumferential direction **e**_c_, b_i_’s and c_i_’s were constant constitutive model parameters. Constitutive model II enhanced model I by allowing explicit coupling in the anistropic term

2 $$\begin{aligned} & \psi_{2} = \frac{1}{2}\left[ {{\text{b}}_{1} \left( {\exp \left[ {{\text{b}}_{2} \left( {{\text{I}}_{1} - 3} \right)^{2} } \right] - 1} \right) + {\text{c}}_{0} \left( {\exp [{\text{Q}}] - 1} \right)} \right] \\ & {\text{Q}} = {\text{c}}_{1} {\text{E}}_{{\text{c}}}^{2} + {\text{c}}_{2} {\text{E}}_{{\text{r}}}^{2} + {\text{c}}_{3} {\text{E}}_{\phi }^{2} + 2{\text{c}}_{4} {\text{E}}_{{\text{c}}} {\text{E}}_{{\text{r}}} \\ \end{aligned}$$

in which E_c_ = **e**_c_ **Ee**_c_ and E_r_ = **e**_r_ **Ee**_r_ were normal components of the Green-Lagrange strain tensor **E** = (**C** *−* **I**)*/*2 along the circumferential and radial directions **e**_c_*,* **e**_r_, respectively. And E_*φ*_ = **e**_c_ **Ee**_r_ was the shear component. Here, c_3_ and c_4_ characterized the contribution of shear and coupling in the strain energy density. Model III included higher order terms in the power series

3 $$\begin{aligned} & \psi_{3} = \frac{1}{2}\left[ {{\text{b}}_{1} \left( {\exp \left[ {{\text{b}}_{2} \left( {{\text{I}}_{1} - 3} \right)^{2} } \right] - 1} \right) + {\text{c}}_{0} \left( {\exp [{\text{Q}}^{\prime } ] - 1} \right)} \right] \\ & {\text{Q}}^{\prime } = {\text{c}}_{1} {\text{E}}_{{\text{c}}}^{2} + {\text{c}}_{2} {\text{E}}_{{\text{r}}}^{2} + {\text{c}}_{3} {\text{E}}_{\phi }^{2} + 2{\text{c}}_{4} {\text{E}}_{{\text{c}}} {\text{E}}_{{\text{r}}} + {\text{c}}_{5} {\text{E}}_{{\text{c}}}^{4} + {\text{c}}_{6} {\text{E}}_{{\text{r}}}^{4} + {\text{c}}_{7} {\text{E}}_{\phi }^{4} + 2{\text{c}}_{8} {\text{E}}_{{\text{c}}}^{2} {\text{E}}_{{\text{r}}}^{2} \\ \end{aligned}$$

In leaflet subregion method iii, each leaflet was split into the coaptation and belly regions. In both subregions, the same constitutive model was used with independent model parameters except for b_1_*,* b_2_ which accounted for the ground matrix contribution. The demarcation between the coaptation and belly regions was estimated from a few fully-coapted mPVs at 10 mmHg along multiple radial cross sections and then interpolated along the circumferential direction (Fig. 1D, Supplementary Table S4). To enforce incompressibility, additional kinematics, i.e. J = 1, were assumed in models I—III in which J represented the Jacobian of the deformation gradient.

#### Optimization

We used the particle swarm optimization method to optimize the parameters of the three constitutive models. For each optimization scheme, 100 iterations were used with cognitive parameter = 0.5, social parameter = 0.3 and swarm inertia = 9. Each particle represented one set of constitutive model parameters whose position was updated iteratively based on current particle and swarm fitness. Random perturbation was included to avoid trapping in a local minimum. In-house Python code was developed to communicate between the IGA solver and PySwarms backend^41^. For each particle, the same cost function as for developing the representative geometry (see Supplementary Methods S1 online) was used to evaluate the weighted mismatch in free edge length, leaflet surface area and cross sectional-profiles between the simulated and representative geometry. Optimization was performed on a 48-core System76 desktop using 20 processors and 100 iterations. Typical runtime for one optimization session was around 2.5 days.

#### ℓ_2_ error

For a point **P** on the simulated mPV geometry, we found the closest point **P**^*′*^ on the representative geometry. The normalized *ℓ*_2_ error at **P** was defined as the distance between **P** and **P**^*′*^ normalized by the diameter of the referential state geometry on the ANL plane (i.e., approximately 1200 µm).

##### Simulated planar biaxial responses

Given the optimal constitutive model parameters, we generated the regional mechanical responses of mPV leaflets in a planar biaxial stretch test in which the mean fiber direction (i.e., the circumferential direction of a leaflet) was aligned with one of the stretching directions (Fig. 6A). Equal loads were applied on the four sides to compute the stretch ratios according to the analytical expressions (see Supplementary Methods S1 online). Average mPV responses were generated using the average constitutive model parameters of the four leaflet subregions based on scheme II—iii (see Supplementary Table S2 online). Previously published porcine^20^ and ovine^21^ planar biaxial data were utilized.

### Supplementary Information

Supplementary Information.

## Data Availability

The data that supports the findings of this study is available from the corresponding author upon reasonable request.

## Abbreviations

mPV: Murine pulmonary heart valve
TVP: Transvalvular pressure
gQOI: Geometric quantities of interest
FE: Free edge
BA: Basal attachment
CA: Commissure attachment
ANL: Annulus
STJ: Sino-tubular-junction
cc/rc: Central circumferential/radial cross sections
