# Supplementary material for: Functional mechanical behavior of the murine pulmonary heart valve
Source: Sci Rep. 2023 Aug 8;13:12852. doi: 10.1038/s41598-023-40158-w (PMC10409802; doi:10.1038/s41598-023-40158-w)
Supplement: Supplementary file 1 — Supplementary Information. [file 41598_2023_40158_MOESM1_ESM.docx]

# SUPPLEMENTARY INFORMATION

### Construction of the generic geometric model

The generic geometric model describing the midsurface geometry of mPV leaflets was constructed in three steps. First, the base model was developed assuming that 1) the ANL and STJ planes of the mPV were parallel and 2) the pulmonary root was cylindrical. Then, two sequential transformations were applied to introduce the nonzero tilt angle between the ANL and STJ planes and trefoil-shaped cross sections of the root. Numerically, the leaflet geometry was constructed as NURBS surfaces using the open-source Python library igakit [(https://www](http://www.openhub.net/p/igakit)).openhub[.net/p/igakit).](http://www.openhub.net/p/igakit))

**Base model**. To illustrate, the construction of a single leaflet that was symmetric with respect to the x-z plane was shown (Fig. [S1](#_bookmark11)). First, let the ANL and STJ planes be two parallel planes with distance H on which the two vertically-aligned circles indicated the ANL and STJ circular guides, respectively, with centers **P**_o_(0*,* 0*,* 0), **P**_s_(0*,* 0*,* H) and radii R_ANL_, R_STJ_ (Fig. [S1](#_bookmark11)A-B). Both commissure points of the leaflet were assumed to locate on the STJ circular guide while the basal attachment was tangent to the ANL circular guide at its midpoint. Next, we introduced the “key points” of the leaflet geometry which included the commissure points

**P**_1_ R_STJ_ cos( θspan )*,* R_STJ_ sin( θspan )*,* H (S1)

2

2

**P**_2_ R_STJ_ cos( θspan )*, −*R_STJ_ sin( θspan )*,* H *,* (S2)

2 2

the lower ends of the commissure lines

**P**_c1_ R_ANL_ cos( θspan )*,* R_ANL_ sin( θspan )*,* H_c_ (S3)

2

2

**P**_c2_ R_ANL_ cos( θspan )*, −*R_ANL_ sin( θspan )*,* H_c_ (S4)

2

2

and the midpoint of the basal attachment **P**_4_(R_ANL_*,* 0*,* 0). Here, θ_span_ was the leaflet angle span and H_c_ *<* H was the height of the lower ends of the commissure lines (Fig. [S1](#_bookmark11)B-D).

The free edge *ℓ*_FE_ was defined as an order-3 NURBS curve with the knot vector being 0*,* 0*,* 0*, χ*_f_*,* 1 *−χ*_f_*,* 1*,* 1*,* 1 and the set of control points being **P**_1_*,* **P**_1_*,* **P**_t_*,* **P**_2_*,* **P**_2_ (Fig. [S1](#_bookmark11)D). Here, 0 *< χ*_f_ *<* 0*.*5 was the free edge shape coefficient and **P**_t_(*δ*_t_*_,_*_x_*, δ*_t_*_,_*_y_*,* H_t_) was an auxiliary point shared by all three leaflets. Denote the midpoint of line **P**_1_**P**_2_ as **P**_n_. Then, the acute angle between lines **P**_n_**P**_s_*,* **P**_n_**P**_t_ characterized the orientation of the plane that the free edge resided in.

The central radial cross section of the leaflet was defined as an order-3 NURBS curve with the knot vector being 0*,* 0*,* 0*,* 1*,* 1*,* 1 and the set of control points being **P**_m_*,* **P**_b_*,* **P**_4_ (Fig. [S1](#_bookmark11)E). Here, **P**_m_ was the midpoint of the free edge, and **P**_b_ was an auxiliary point defined by the angle θ_b_ between lines **P**_4_**P**_b_*,* **P**_4_**P**_o_ and its distance to **P**_4_ which was determined by

*||***P**_4_**P**_b_*||* = (*χ*_b_ + 1)*||***P**_4_**P**_o_*||.* (S5)

Here, *χ*_b_ *> −*1 was the belly shape coefficient.

The basal attachment *ℓ*_BA_ connected **P**_c1_*,* **P**_4_*,* **P**_c2_ (Fig. [S1](#_bookmark11)E). With the assumption that the pulmonary

artery was cylindrical with radius R_ANL_, the basal attachment was parameterized by

x(t) = R_ANL_ cos( θspan t)*,* y(t) = R_ANL_ sin( θspan t)*,* z(t) = H_c_t^2^ (S6)

2 2

in which *−*1 *≤* t *≤* 1.

Given the skeleton of the geometry, we defined the interior of the surface by introducing the master control curve *ℓ*_M_. The idea came from the observation that the commissure line **P**_1_**P**_c1_ was a degenerated order-3 NURBS curve with the knot vector being 0*,* 0*,* 0*,* 1*,* 1*,* 1 and the set of control points being **P**_1_*,* **P**_cm1_*,* **P**_c1_ in which **P**_cm1_ was the midpoint of **P**_1_**P**_c1_. Same for the other commissure line **P**_2_**P**_c2_. Therefore, both followed the same rule of construction as the central radial cross section. For convenience, we used an order-3 NURBS curve to connect the middle control points (i.e., **P**_cm1_*,* **P**_b_*,* **P**_cm2_) and formed the master control curve. The master control curve allowed us to form any radial cross sections together with the free edge and basal attachment (Fig. [S1](#_bookmark11)F). Particularly, for 0 *≤* t*^′^ ≤* 1, the associated radial cross section was an order-3 NURBS curve with the knot vector being 0*,* 0*,* 0*,* 1*,* 1*,* 1 and the set of control points being *ℓ*_FE_(t*^′^*)*, ℓ*_M_(t*^′^*)*, ℓ*_BA_(2t*^′^ −* 1).


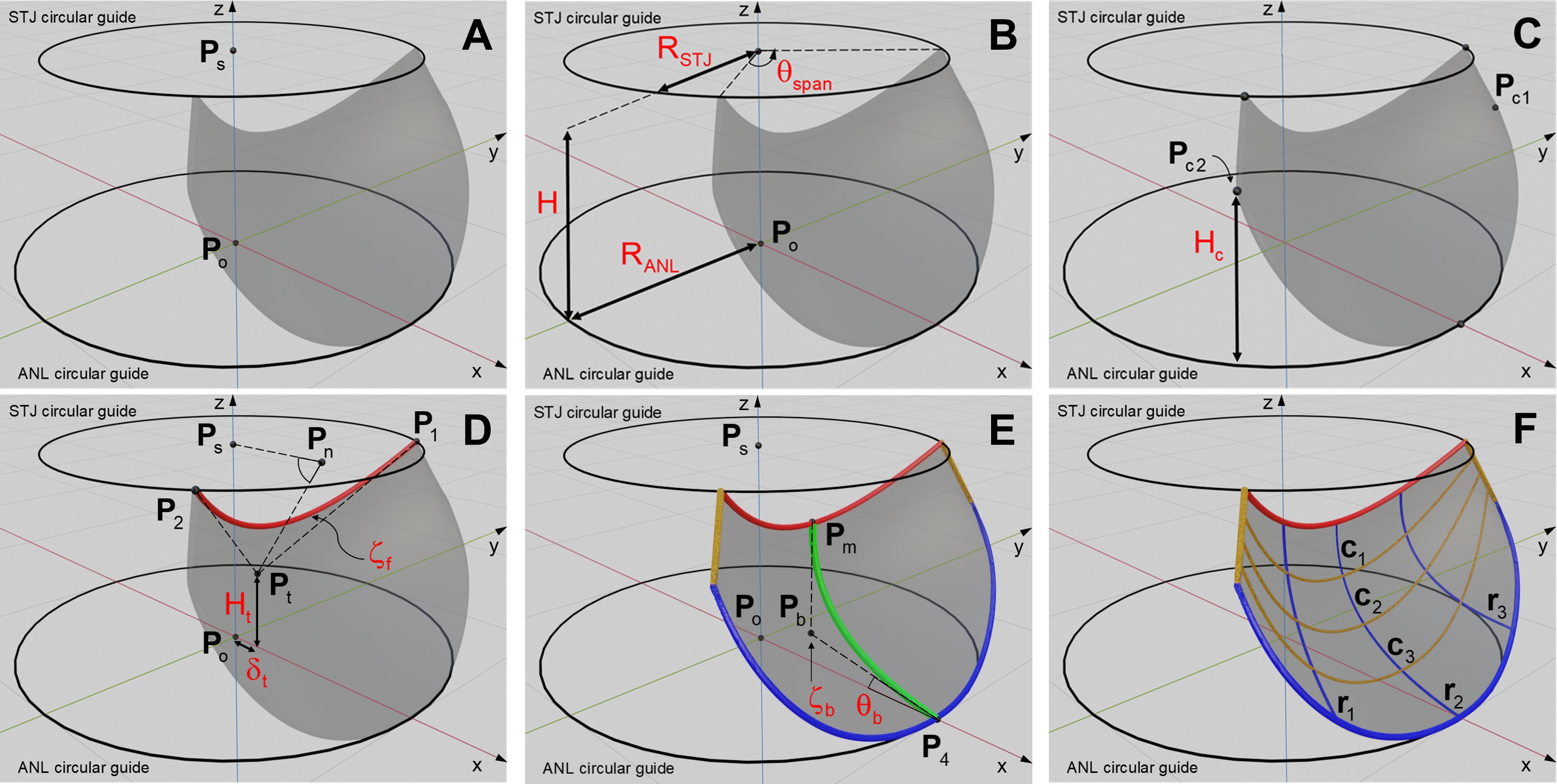


**Figure S1.** Schematic for the base generic geometric model. (A) showed the outline of a leaflet to be constructed and the two circular guides. (B-C) illustrated key geometric parameters R_ANL_*,* R_STJ_*,* H*,* θ_span_*,* H_c_. (D-E) illustrated the construction of the free edge and central radial cross section, respectively. (F) showed the quantile cross-sections in the circumferential and radial directions.

**Full model**. In the full geometric model, two transformations *M*_t_*, M*_b_ were applied to introduce the trefoil-shaped root cross section and nonzero tilt angle between ANL and STJ planes. In the cylindrical

coordinate, we have

rcosθ

*M*_t_ : rsinθ *'→* rsinθ *·* k(θ) *,*

rcosθ *·* k(θ)

z

  

rcosθ

M*_b_* : rsinθ *'→* 

rcosθsin(z*/*ρ) +ρsin(z*/*ρ)

z

z

rcosθcos(z*/*ρ)



rsinθ  *,*

where ρ = H*/*β and k(θ) was the scalar trefoil function satisfying the following conditions

k(*±*θspan ) = 1*,*

2

k(0) = ktrefoil*.*

For simplicity, we chose

k(θ) = (k_trefoil_ *−* 1) 1 *−* ( 2θ )^2^ + 1*.*

θspan

Both transformations were applied to the key point positions (i.e., **P**_1_*,* **P**_2_*,* **P**_c1_*,* **P**_c2_*,* **P**_4_) and basal attachment to displace the leaflet boundary. Then, the same construction steps detailed in the base model were applied to create the free edge, central radial cross section and the interior leaflet surface. Note, given the right and left symmetry of the valve, those leaflet-level parameters shared the same values with the right and left leaflets. Additionally, we noticed that the tilt pattern of STJ plane also largely observed right and left symmetry. Hence there was only one anglular degree of freedom present in *M*_b_. The full list of model parameters were summarized in Table [S1](#_bookmark12).

**Table S1.** List of parameters in the full generic geometric model.

| **Valve-level** | **Leaflet-level** |
| --- | --- |
| R_ANL_ radius of ANL circular guide  R_STJ_ radius of STJ circular guide H valve height  H_c_ height of the commissure line at lower end  β tilt angle between ANL and STJ planes H_t_ height of **P**_t_  *δ*_t_*_,_*_x_*, δ*_t_*_,_*_y_ offset of **P**_t_ | θ_span_ leaflet angle span  θ_b_ tangent angle at belly  *ζ*_b_ belly shape parameter  *ζ*_f_ free edge shape parameter k_t_ trefoil parameter  w leaflet thickness |

### Construction of individual mPV geometric model

The cost function *φ* for fitting the individual mPV geometric model was given below:

*φ* = f_1_ +f_2_

f_1_ = ∑

leaflet*∈{*A*,*R*/*L*}*

w_0_ D(c_2_*,* cˆ_2_) +D(r_2_*,* ˆr_2_)

f_2_ = ∑

_i_

p*∈{*Lp*,*LFE*,*LBA*,*LCA*,*dnod*,*dcomm*,*a*ℓ}*

w p *−* pˆ

in which c_2_*,* r_2_ were the central (projected) cross sectional profiles along circumferential and radial directions, D(*·,·*) was a function evaluating the mean square distance between corresponding points of two planar curves , symbols with ˆindicated gQOI derived from a trial geometry; otherwise, directly measured gQOI were used. w_i_ were weights manually set from case to case. For conciseness, valve-level and leaflet-level gQOI were not differentiated.

### Construction of referential state geometric model

The cost function *φ* for fitting the referential geometric model was given below:

*φ* = ∑

p*∈{*Lp*,*LFE*,*LBA*,*LCA*,*a*ℓ}*

w p *−* pˆ

Symbols withˆindicated gQOI derived from a trial geometry; otherwise, directly measured gQOI were used. For L_p_*,* L_FE_*,* L_BA_*,* L_CA_*,* A*_ℓ_*, the value of the weight was equal to 10*,* 10*,* 10*,* 1*,* 10*^−^*^4^, respectively.

_i_

### Construction of representative geometric model

The cost function *φ* for fitting the representative mPV geometry was given below:

*φ* = f_1_ +f_2_

f_1_ = ∑

leaflet*∈{*A*,*R*/*L*}*

w_0_ D(c_2_*,* cˆ_2_) +D(r_2_*,* ˆr_2_)

f_2_ = ∑

_i_

p*∈{*LFE*,*a*ℓ}*

w p *−* pˆ

Symbols withˆindicated gQOI derived from a trial geometry; otherwise, directly measured gQOI were used. For L_FE_ and a*_ℓ_*, the value of the weight was equal to 10*,* 10*^−^*^4^, respectively.

### Construction of root distention model

Let t = TVP*/*30mmHg denote the normalized TVP, and **P**(X_1_*,* X_2_*,* X_3_) be a point on the basal and commis- sure attachment at the referential state. The components of displacement along lab coordinates u_1_*,* u_2_*,* u_3_ under increasing TVP were described by the following model

u_1_(X_1_*,* X_2_*,* X_3_*,* TVP) = f(θ) cos(θ)tin-plane u_2_(X_1_*,* X_2_*,* X_3_*,* TVP) = f(θ) sin(θ)tin-plane u_3_(X_1_*,* X_2_*,* X_3_*,* TVP) = g(X_3_*,* θ)tout-of-plane

in which f*,* g were the magnitude of displacement in the X_1_ *−* X_2_ and X_3_ directions at 30 mmHg, and

t = exp(*−*1*/*t)

in-plane

exp(*−*1*/*t) +exp(*−*1*/*(2*/*3 *−* t))

t = exp(*−*1*/*t)

out-of-plane

exp(*−*1*/*t) +exp(*−*1*/*(1 *−* t))

were functions describing the rate of root distention as TVP increased.

Functions f*,* g were defined as follows

f(θ) = 4 ur*,*mid *−* ur*,*comm (θ *−* θmin)(θmax *−* θmin) +ur*,*comm

(θmax *−* θmin)2

(uz*,*mid(1 *−* X3 )*,* H = θ*−*θmin Hc*,*max + θmax*−*θ Hc*,*min on BA

g(θ*,* X_3_) =

H

θmax*−*θmin

θmax*−*θmin

0 on CA

in which θ_min_*,* θ_max_ were the maximum and minimum angles for the leaflet. H_c_*_,_*_min_*,* H_c_*_,_*_max_ were the height of the commissure points on the commissure attachment at the referential state. u_r_*_,_*_mid_*,* u_r_*_,_*_comm_*,* u_z_*_,_*_mid_ were control parameters defining the radial distention at midpoint of the basal attachment (red arrows), radial distention at the commissure point (blue arrows) and vertical distention at mid point of the basal attachment (green arrows); see Fig. [S5](#_bookmark13).


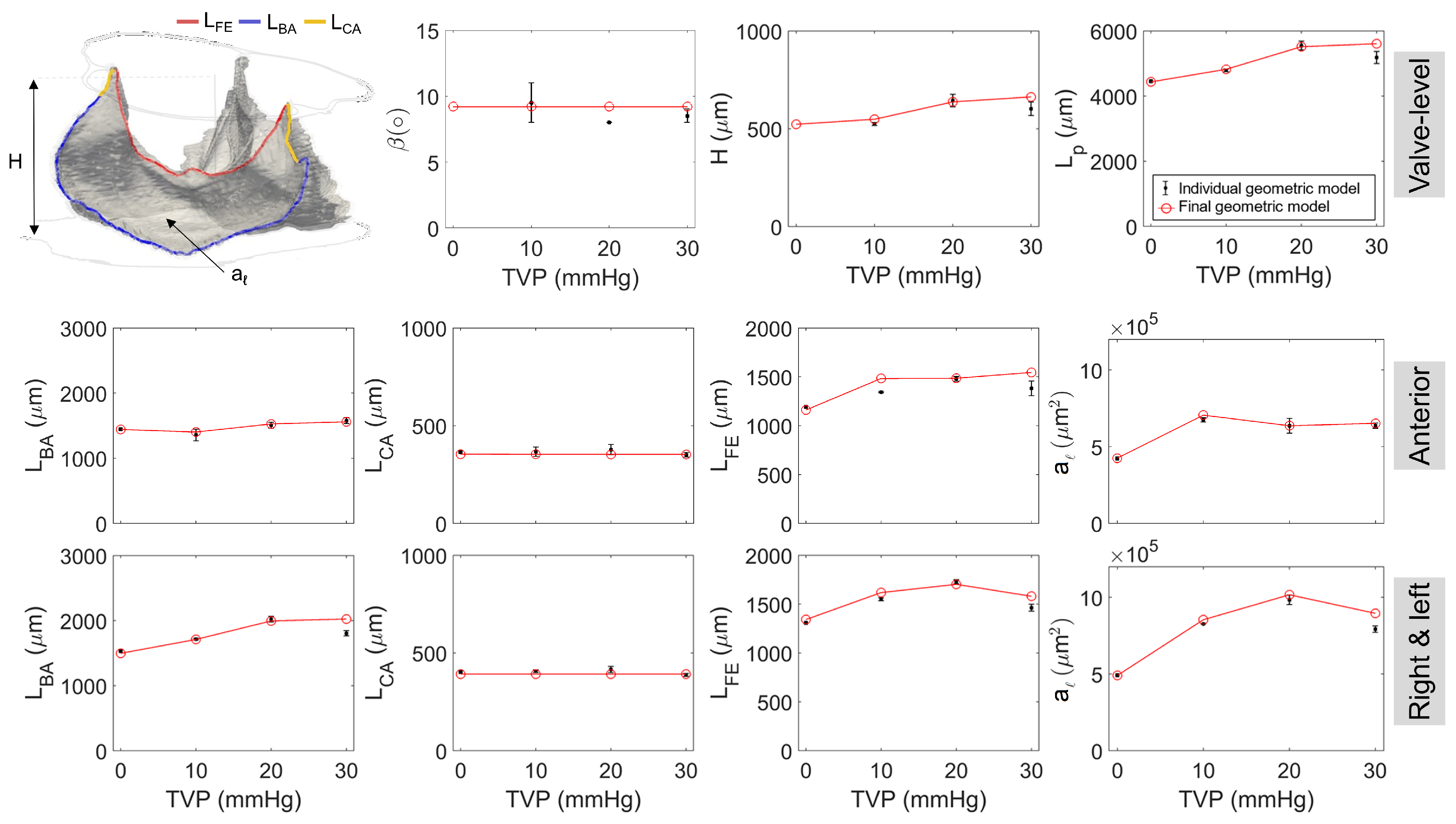


**Figure S2.** Key gQOI derived from the final geometric model (red lines) vs. individual geometric model (black symbols, mean*±*s.e.m) indicated reasonable agreement. Due to mPV leaflet collapse, no individual geometric model was derived at 0 mmHg. Hence, corresponding data points represented direct measurement from *µ*CT images. However, for tilt angle β and valve height H, direct measurement was not available either thus not shown in the plots.


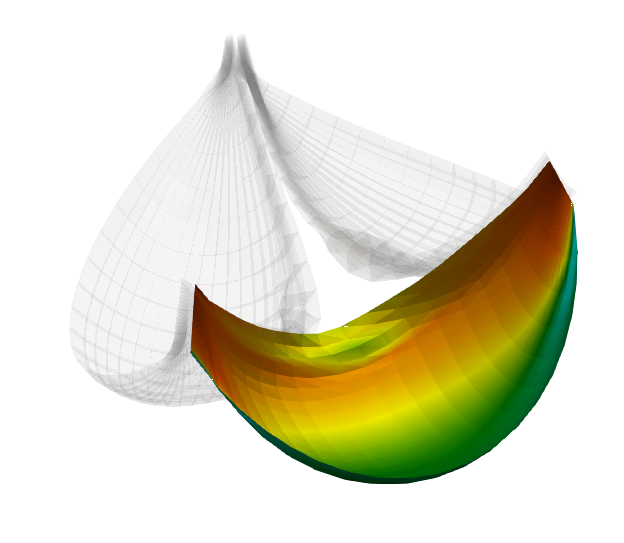

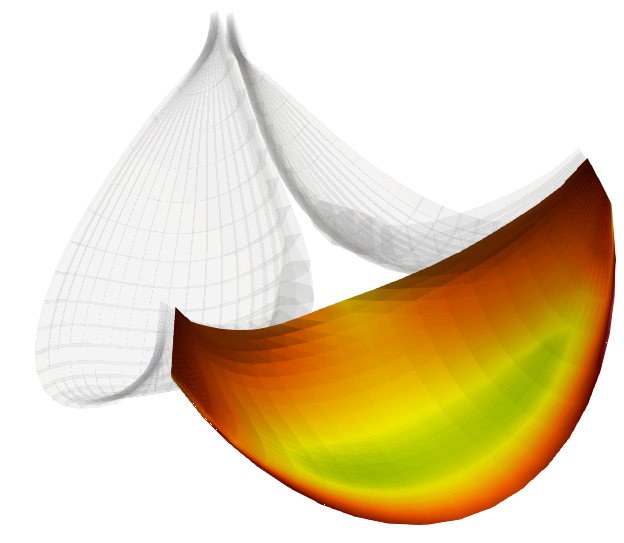

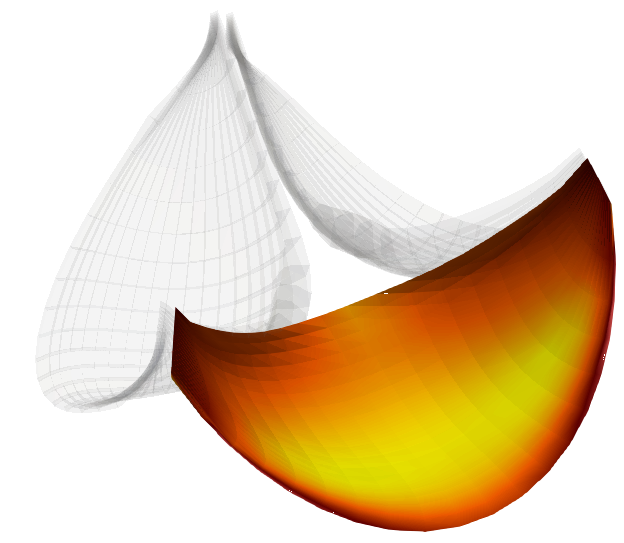

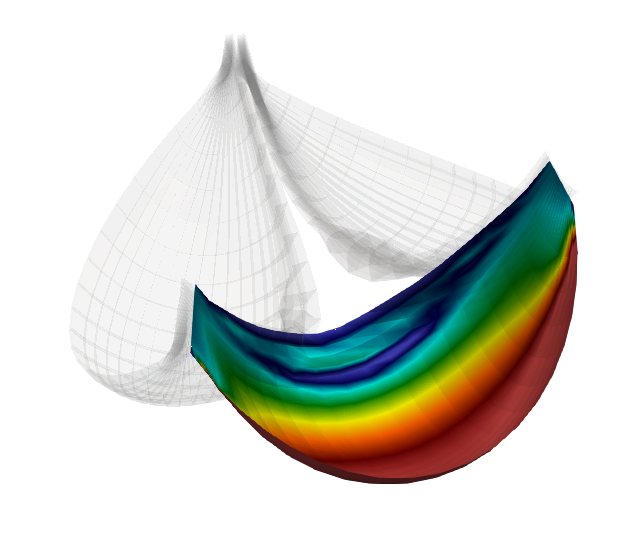

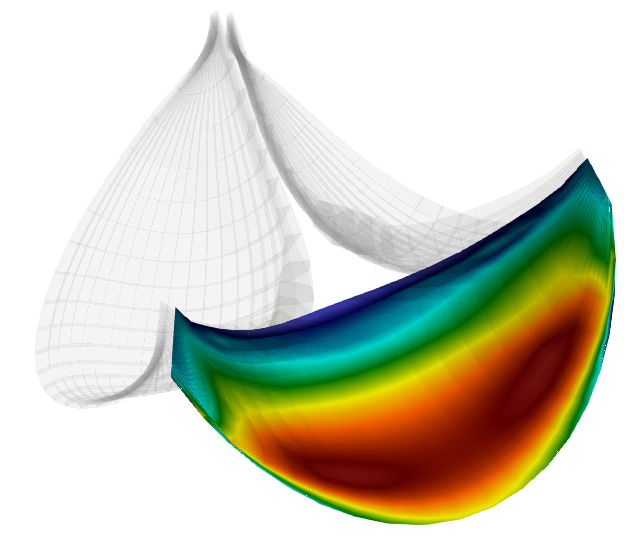

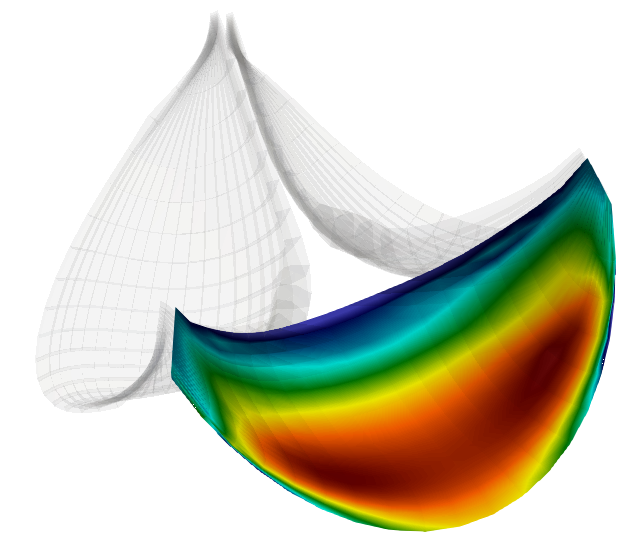

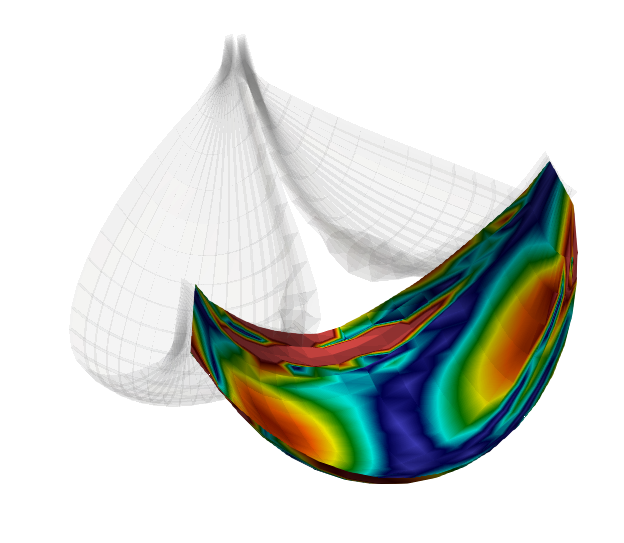

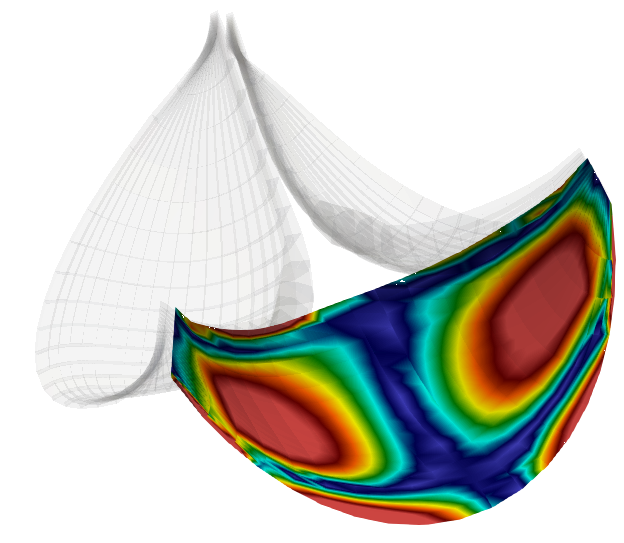

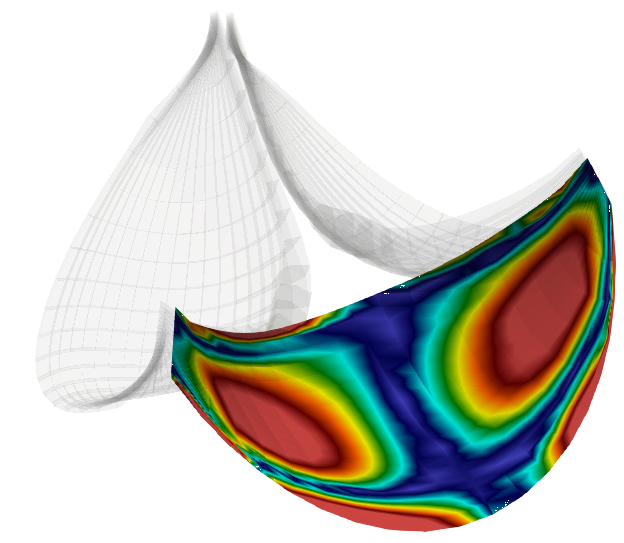
10 mmHg 20 mmHg 30 mmHg


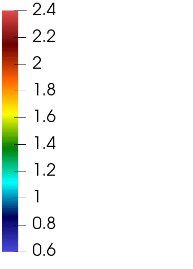


*λ*_r_


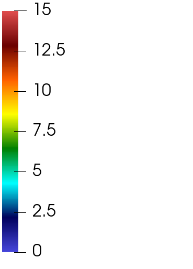


∆θ_cr_ (*^◦^*)


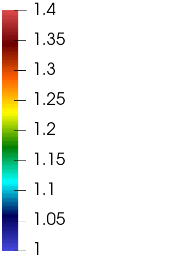


*λ*_c_

**Figure S3.** The simulated mPV geometries at 10, 20 and 30 mmHg based on the optimization scheme II - iii color coded by *λ*_c_*, λ*_r_*,* ∆θ_cr_.


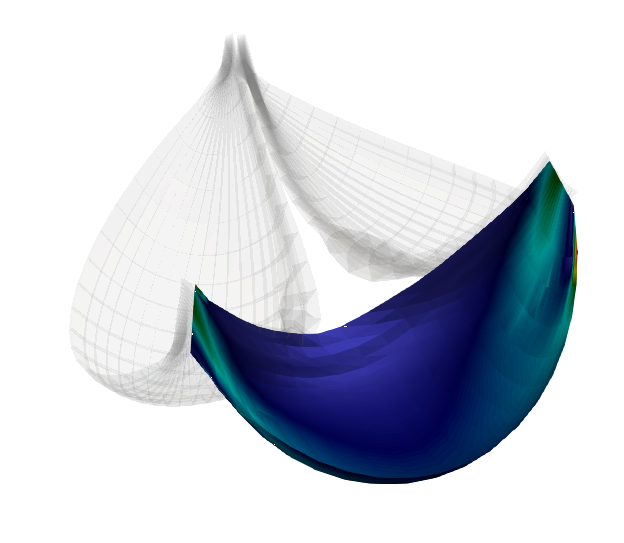

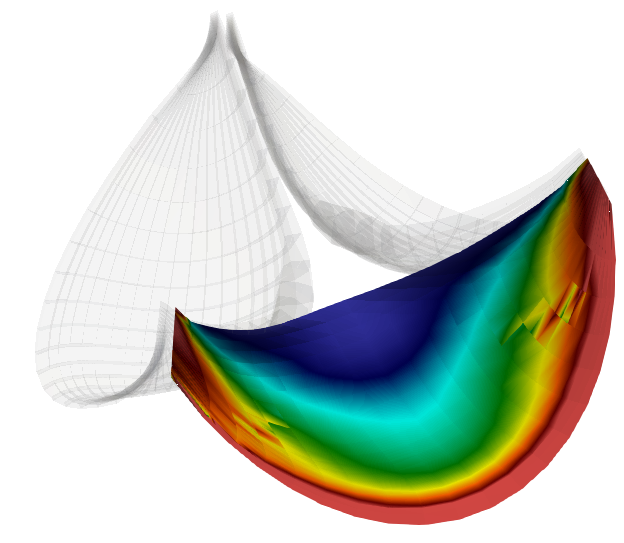

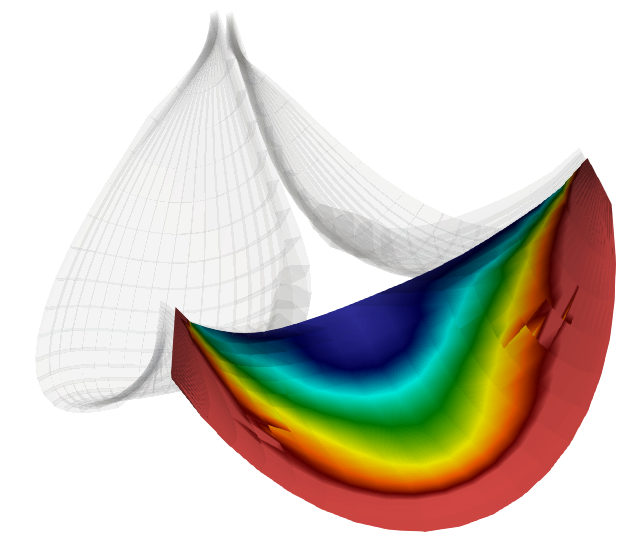

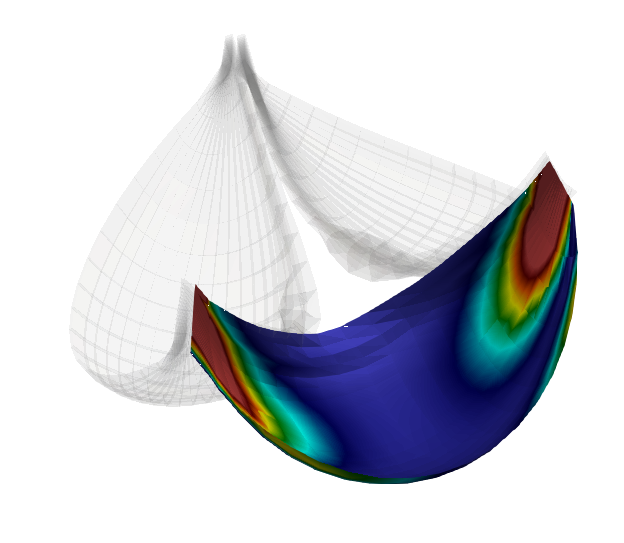

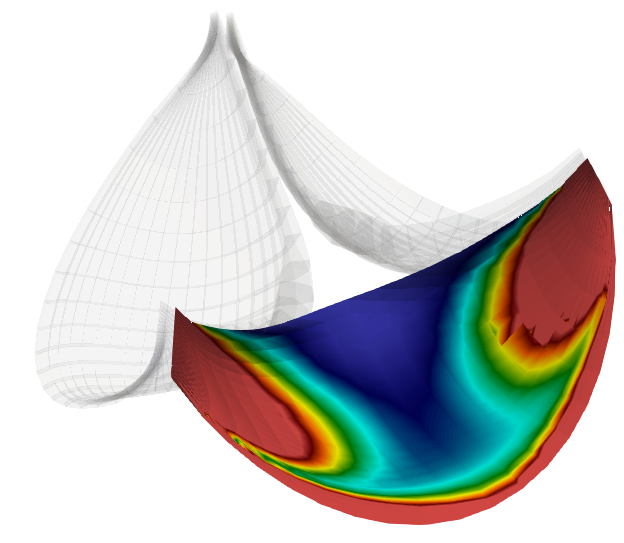

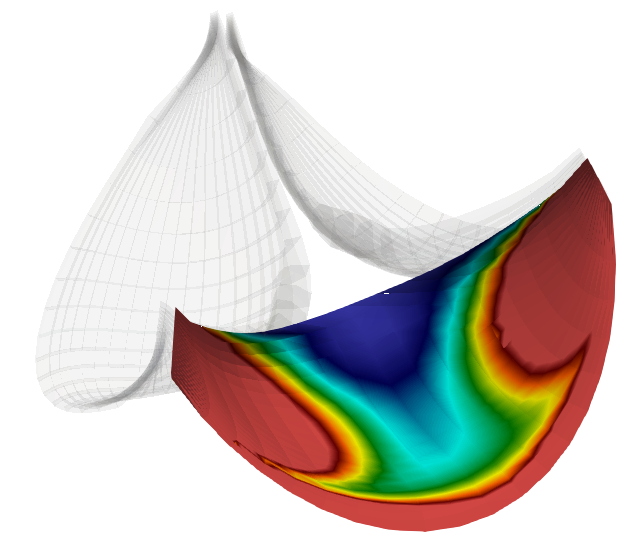

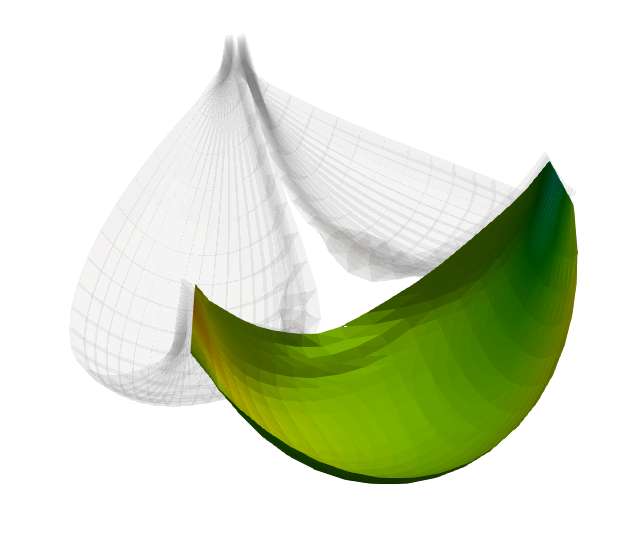

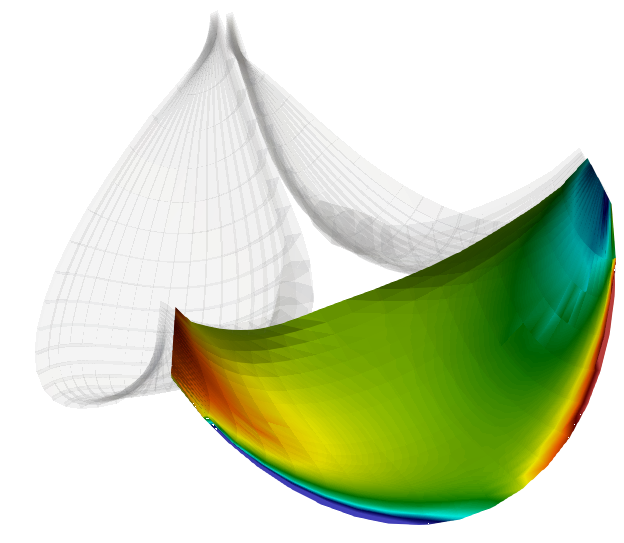

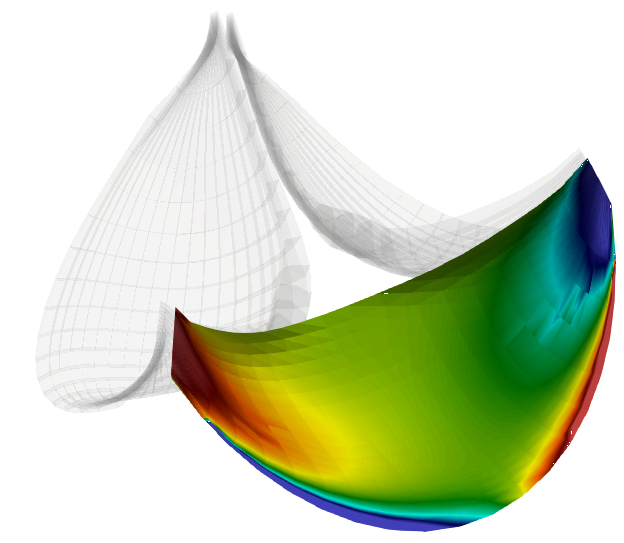
10 mmHg 20 mmHg 30 mmHg


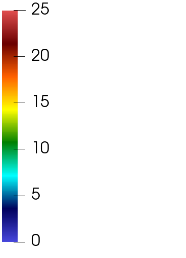


S_rr_ (kPa)


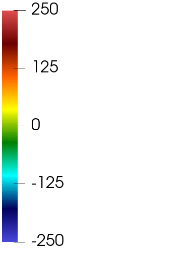


S_cr_ (kPa)


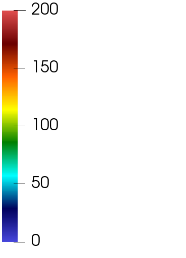


S_cc_ (kPa)

**Figure S4.** The simulated mPV geometries at 10, 20 and 30 mmHg based on the optimization scheme II - iii color coded by S_cc_*,* S_rr_*,* S_cr_.


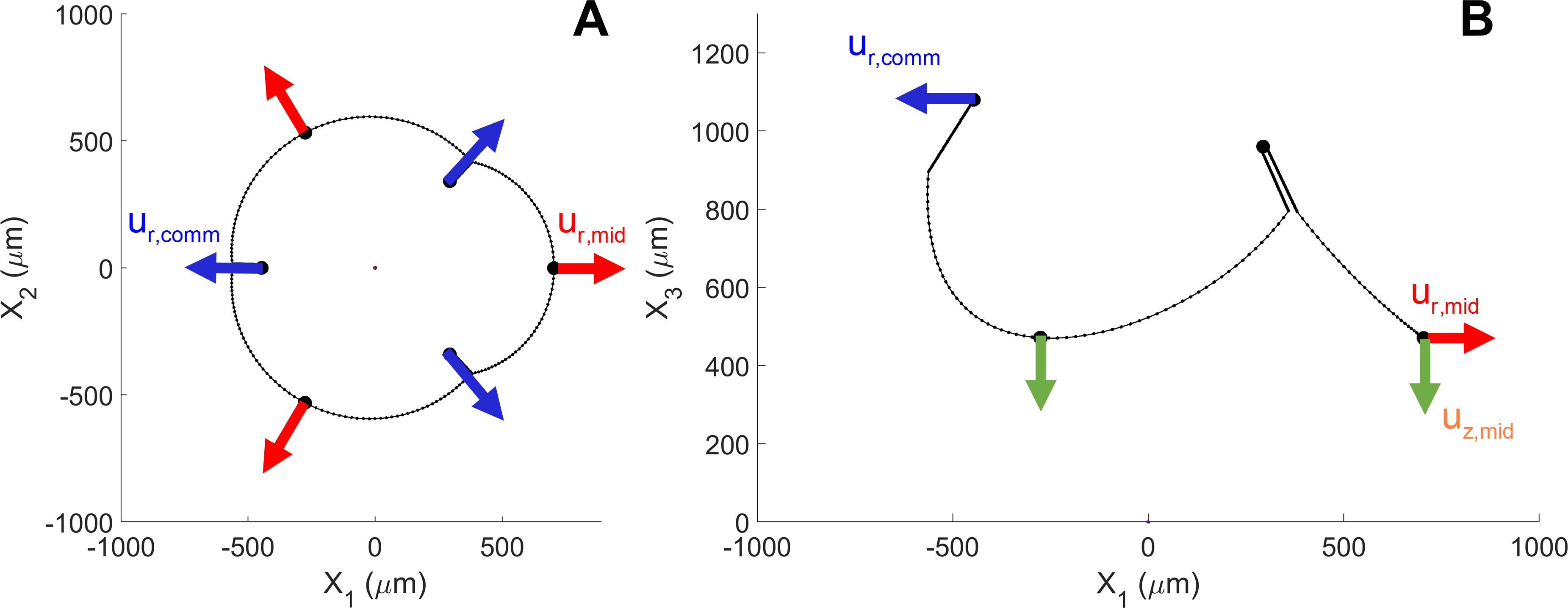


**Figure S5.** Key control parameters in the root distention model from the top (A) and side (B) views. The black lines indicated the total attachment of the referential state geometry at 0 mmHg.

**Table S2.** Estimate demarcation (mean*±*s.e.m, n=3) between the coaptation and belly regions. *η* was the ratio between the arc-length from the demarcation point to the BA end of a radial cross section and the entire cross section length. The circumferential position indicated the position of a radial cross section. For example, 1/2 = central slice. 1/6 = a slice closer to the commissure attachment.

| circumferential position | 1/6 | 2/6 | 1/2 |
| --- | --- | --- | --- |
| *η* | 56*±*4% | 81*±*2% | 85*±*1% |

### Derivation of the mechanical response under planar biaxial loading

For constitutive model I,

*∂ψ*_1_(I_1_*,* I_4_)

*∂ψ*_1_

*∂ψ*_1_

*∂* **E** = 2 *∂* I1 **I** + 2 *∂* I4 **e**_c_ *⊗* **e**_c_*,*

*∂ψ*_1_

*∂* I_1_

*∂ψ*_1_

*∂* I_4_

*∂ψ*_1_

= b_1_b_2_(I_1_ *−* 3) exp[b_2_(I_1_ *−* 3)^2^]*,*

= c_0_c_1_(I_4_ *−* 1) exp[c_1_(I_4_ *−* 1)^2^]*,*

*∂ψ*_1_

*∂* E_33_ = 2 *∂* I_1_ *.*

knowing that *^∂^*^I^1 = 2**I***, ^∂^*^I4^ = 2**e**_c_ *⊗* **e**_c_*,* **e**_3_ *·* **e**_c_ = 0. For constitutive model II,

*∂* **E** *∂* **E**

*∂ψ*_2_(I_1_*,* Q) *∂ψ*_2_ *∂ψ*_2_ *∂* Q

*∂* **E** = 2 *∂* I1 **I** +

*∂* Q *∂* **E** *,*

*∂ψ*_2_

*∂* I_1_

*∂ψ*_2_

= b_1_b_2_(I_1_ *−* 3) exp[b_2_(I_1_ *−* 3)^2^]*,*

1

*∂* Q =

*∂* Q

2 c_0_ exp(Q)*,*

2c_1_E_c_ + 2c_4_E_r_ 2c_3_E*_φ_* 0

*∂* **E** = 



2c_3_E*_φ_* 2c_2_E_r_ + 2c_4_E_c_ 0 *.*

0 0 0

knowing that Q = c_1_E^2^ +c_2_E^2^ +c_3_E^2^ + 2c_4_E_c_E_r_. The expressions for model III were similar.

c r *φ*

**Table S3.** Optimal constitutive model parameters based on scheme II - ii. Mean*±*s.e.m. were reported for the average column which combined the optimal constitutive model parameters in all four leaflet subregions.

| Parameters for constitutive model II | Right & Left | | Anterior | | Average |
| --- | --- | --- | --- | --- | --- |
|  | Coapt | Belly | Coapt | Belly |  |
| b_1_ (Pa) | 24.61 | 24.61 | 33.73 | 33.73 | 29.17 *±* 2.63 |
| b_2_ | 0.084 | 0.084 | 0.97 | 0.97 | 0.53 *±* 0.26 |
| c_0_ (Pa) | 20.14 | 30.68 | 187.09 | 11.94 | 63.46 *±* 41.48 |
| c_1_ | 42.44 | 42.64 | 44.79 | 43.14 | 43.25 *±* 0.53 |
| c_2_ | 0.65 | 0.083 | 7.58 | 1.19 | 2.38 *±* 1.75 |
| c_3_ | 3.29 | 2.35 | 4.45 | 3.82 | 3.48 *±* 0.44 |
| c_4_ | 2.96 | 3.40 | 4.45 | 3.55 | 3.59 *±* 0.31 |

Consider a square thin sheet made of mPV material under biaxial stretching**^?^**^,^ [^43^](#_bookmark50) For simplicity, it was assumed that the fiber and perpendicular directions (i.e., **e**_c_*,* **e**_r_) of the material aligned with the stretch directions. Denote the stretch ratios along **e**_c_*,* **e**_r_ as *λ*_c_*, λ*_r_, respectively. Assuming incompressibility, the

associated deformation gradient was hence given by

*λ*_c_ 0 0



**F** = 0 *λ*_r_ 0 

*.*

 0 0 

1

*λ*c*λ*r

Let the potential energy density of the material be *ψ*ˆ = *ψ*(**F**) *−* p(J *−* 1) in which *ψ* was a generic form of potential energy, and J = det(**F**) was the Jacobian determinant of the mapping from the reference to current configuration. Knowing that J = 1 and *^∂^*^J^ = J**F***−*T, the PK2 stress was

*∂* **F**

**S** = *∂ψ −* p**C***^−^*^1^

*∂* **E**

With the plane stress assumption, i.e., S_33_ = 0, we had

p = C_33_ *∂ψ ,* thus **S** = *∂ψ −* C_33_ *∂ψ* **C***^−^*^1^*.*

*∂* E_33_ *∂* **E** *∂* E_33_
